# Direct repression of MYB by ZEB1 suppresses proliferation and epithelial gene expression during epithelial-to-mesenchymal transition of breast cancer cells

**DOI:** 10.1186/bcr3580

**Published:** 2013-11-27

**Authors:** Honor J Hugo, Lloyd Pereira, Randy Suryadinata, Yvette Drabsch, Thomas J Gonda, N P A Devika Gunasinghe, Cletus Pinto, Eliza TL Soo, Bryce JW van Denderen, Prue Hill, Robert G Ramsay, Boris Sarcevic, Donald F Newgreen, Erik W Thompson

**Affiliations:** 1 Invasion and Metastasis Unit, St Vincent’s Institute, 9 Princes St, Fitzroy 3065, Victoria, Australia; 2 Differentiation and Transcription Laboratory, Peter MacCallum Cancer Centre, St Andrews Place, East Melbourne 3002, Victoria, Australia; 3 Sir Peter MacCallum Department of Oncology, The University of Melbourne, Parkville, Australia; 4 Cell Cycle and Cancer Unit, St Vincent’s Institute, Melbourne, Australia; 5 University of Queensland Diamantina Institute, Translational Research Institute, 37 Kent St, Woolloongabba 4102, Queensland, Australia; 6 University of Melbourne Department of Surgery, St. Vincent’s Hospital, 41 Victoria Pde, Fitzroy 3065, Victoria, Australia; 7 Embryology Laboratory, Murdoch Children’s Research Institute, Royal Children’s Hospital, 50 Flemington Rd, Parkville 3052, Victoria, Australia; 8 University of Melbourne Department of Medicine, St. Vincent’s Hospital, Melbourne, Australia; 9 University of Melbourne Department of Pathology and St. Vincent’s Pathology, Melbourne, Australia; 10 Department of Anatomy, Faculty of Medicine, University of Peradeniya, Peradeniya, Sri Lanka; 11 School of Pharmacy, University of Queensland, PACE, 20 Cornwall Street, Woolloongabba 4102, Queensland, Australia; 12 Present address: Edinburgh Cancer Research UK Centre, MRC Institute of Genetics and Molecular Medicine, University of Edinburgh, EH42XR Edinburgh, United Kingdom

## Abstract

**Introduction:**

Epithelial-to-mesenchymal transition (EMT) promotes cell migration and is important in metastasis. Cellular proliferation is often downregulated during EMT, and the reverse transition (MET) in metastases appears to be required for restoration of proliferation in secondary tumors. We studied the interplay between EMT and proliferation control by MYB in breast cancer cells.

**Methods:**

MYB, ZEB1, and CDH1 expression levels were manipulated by lentiviral small-hairpin RNA (shRNA)-mediated knockdown/overexpression, and verified with Western blotting, immunocytochemistry, and qRT-PCR. Proliferation was assessed with bromodeoxyuridine pulse labeling and flow cytometry, and sulforhodamine B assays. EMT was induced with epidermal growth factor for 9 days or by exposure to hypoxia (1% oxygen) for up to 5 days, and assessed with qRT-PCR, cell morphology, and colony morphology. Protein expression in human breast cancers was assessed with immunohistochemistry. ZEB1-MYB promoter binding and repression were determined with Chromatin Immunoprecipitation Assay and a luciferase reporter assay, respectively. Student paired *t* tests, Mann–Whitney, and repeated measures two-way ANOVA tests determined statistical significance (*P* < 0.05).

**Results:**

Parental PMC42-ET cells displayed higher expression of ZEB1 and lower expression of MYB than did the PMC42-LA epithelial variant. Knockdown of ZEB1 in PMC42-ET and MDA-MB-231 cells caused increased expression of MYB and a transition to a more epithelial phenotype, which in PMC42-ET cells was coupled with increased proliferation. Indeed, we observed an inverse relation between MYB and ZEB1 expression in two *in vitro* EMT cell models, in matched human breast tumors and lymph node metastases, and in human breast cancer cell lines. Knockdown of MYB in PMC42-LA cells (MYBsh-LA) led to morphologic changes and protein expression consistent with an EMT. ZEB1 expression was raised in MYBsh-LA cells and significantly repressed in MYB-overexpressing MDA-MB-231 cells, which also showed reduced random migration and a shift from mesenchymal to epithelial colony morphology in two dimensional monolayer cultures. Finally, we detected binding of ZEB1 to MYB promoter in PMC42-ET cells, and ZEB1 overexpression repressed MYB promoter activity.

**Conclusions:**

This work identifies ZEB1 as a transcriptional repressor of MYB and suggests a reciprocal MYB-ZEB1 repressive relation, providing a mechanism through which proliferation and the epithelial phenotype may be coordinately modulated in breast cancer cells.

## Introduction

Epithelial-to-mesenchymal transition (EMT), well described in development [1], enables carcinoma cells to invade local tissues and metastasize to distant sites [2]. EMT causes cell-cell detachment and basement membrane degradation, permitting cell migration aided by actin cytoskeletal rearrangements. EMT triggers myriad intracellular and extracellular signals, which combine to generate motile cells and provide protection against pro-death signals from the host and anticancer therapies, on the journey to secondary sites and while in the systemic circulation (reviewed in [3]).

ZEB1 (zinc-finger E-box-binding homeobox 1) is a dual zinc-finger, DNA-binding transcription factor, recognizing bipartite E-boxes (CACCTG, CAGGTG) and/or Z-boxes (CAGGTA) [4,5]. ZEB1 as with ZEB2, Snail1 and 2, Twist1 and 2, TCF3 and 4, FoxC2, Goosecoid, KLF8 and Id1 orchestrate EMT transcriptional and morphologic changes (reviewed in [6]). In EMT, ZEB1 is a direct transcriptional repressor of E-cadherin [7] plakophilin3 [8], Crumbs3, HUGL2, and Pals1 [9,10]. ZEB1 may also promote metastasis, as shown in a xenograft mouse model [10] and significantly higher ZEB1 expression is seen in human breast cancer cell lines of the more mesenchymal/invasive basal B subgroup [11-13].

The transcription factor MYB is an oncogene in human leukemias, and in epithelial cancers of the colon and breast (reviewed in [14,15]). MYB promotes proliferation and inhibits differentiation [14]. We have shown that MYB drives proliferation and suppresses apoptosis and differentiation in estrogen receptor (ER)-positive breast cancer cells in response to estrogen [16,17], and that it is essential for mammary carcinogenesis in xenograft and transgenic models [18].

Mutual regulatory relations have been defined for MYB and ZEB1 in the hematopoietic system. MYB and Ets-1 synergize to overcome transcriptional repression of MYB by ZEB1 [19], and MYB has been shown to regulate ZEB1 expression in the developing inner ear [20]. Conversely, ZEB1 maintains tight regulatory control over MYB during T-cell differentiation [21]. However, the mechanism of this relation has not been defined, and it has not been reported in a solid tumor (cell) context.

A number of transcriptional repressors of CDH1 have been demonstrated to impede cell-cycle progression directly (reviewed in [22]). Colon cancer cells undergoing an EMT at the invasive front coincide with the region where ZEB1 is expressed [23] and display a downregulation of proliferation [24]. Conversely, miR-200 family members, which target ZEB mRNA for degradation [4], have been shown to have a pro-proliferative role [25,26], thus promoting the growth of breast cancer cell metastases [27]. However, a pro-proliferative role has also been described for ZEB1, because in some contexts, it represses the cell-cycle inhibitors p21 and p73 [28,29]. The current study sought to determine the ZEB1/MYB/proliferation interplay in the epidermal growth factor (EGF)-responsive PMC42 model of breast cancer EMT.

The PMC42 model system [6] comprises the parental cell line PMC42-ET (ET) and its more epithelial variant PMC42-LA (LA). Both lines exhibit EMT in response to EGF [30,31], with marked differences in EMT-marker protein expression and arrangement [32]. Here we have identified an inverse relation between ZEB1 and MYB throughout these cell states, and also in the breast cancer cell lines MDA-MB-231 and MDA-MB-468. We showed that ZEB1 is a key player in promoting the mesenchymal phenotype and regulating the proliferative rate in ET cells through the direct transcriptional repression of MYB. Release of MYB repression promotes an epithelial phenotype, or MET, in which proliferation is restored. MYB and CDH1 gene expression were found to correlate in human breast cancer cell lines and in primary human breast tumors and metastases. Collectively our data link the transcriptional regulation of MYB by ZEB1 to the proliferative state of cells during EMT-MET in breast cancer, and also indicate a contribution of MYB to the epithelial phenotype.

## Methods

### Cell culture

PMC42-ET (ET) cells were derived from a breast cancer pleural effusion by Dr. Robert Whitehead, Ludwig Institute for Cancer Research, Melbourne, Australia, with appropriate institutional ethics clearance (Institutional Review Board of the Peter MacCallum Hospital, Melbourne) and informed consent of the patient. The PMC42-LA (LA) subline was derived further from the parental PMC42- ET cells by Dr. Leigh Ackland, Deakin University, Melbourne, Australia [30,33-35]. These cells, and all modified (shRNA) derivatives, were maintained at 37°C, 5% CO_2_ in RPMI1640 containing 10% fetal calf serum (FCS; Thermo Fisher Scientific, Waltham, MA, USA). T47D cells and MCF-7 cells (used in EGF and hypoxic assays) were obtained from the American Type Culture Collection (ATCC, Rockville, MD, USA). MCF-7 cells used for constitutive MYB expression were provided by the Michigan Cancer Foundation to the Garvan Institute, Sydney, Australia. MDA-MB-468 cells originally from the ATCC were transferred from the Lombardi Cancer Center, Washington, DC, USA. All were maintained in Dulbecco Modified Eagle Medium (DMEM; Thermo Fisher Scientific). For induction of EMT via hypoxia, MDA-MB-468 cells were cultured at 1% O_2,_ 5% CO_2_ in a hypoxia workstation (INVIVO_2_400; Ruskinn Technology Ltd, Bridgend, UK). For EGF-induced EMT, approximately 30% confluent LA cells were treated with EGF (Collaborative Biomedical Products, Bedford, MA, USA) at 10 ng/ml. After trialing incubation times and concentrations we concluded that treatment of PMC42-LA cells with EGF for 9 days generated a robust and reproducible EMT (as indicated by QRT-PCR and morphology; data not shown). Three dimensional (3D) culture conditions were created by using the Matrigel “on-top” assay [36].

### Creation of modified cell lines

Generation of shRNA-expressing ET, LA, MDA-MB-468, or MDA-MB-231 cells was achieved by using the Lenti-X HTX packaging system (Clonetech Laboratories, Mountain View, CA, USA) and following the manufacturer’s instructions; shRNA sequences (Table 1) were encoded within a GFP-expressing lentiviral vector (pGIPZ; Thermo Scientific Open Biosystems, distributed by Millenium Science, Surrey Hills, VIC, Australia). Successfully transduced cells were selected by cell sorting for GFP; thus a transduced pool rather than individual clones was used in these studies. Stable MYBs expressing MDA-MB-231 and MCF-7 cells were generated by lentiviral transduction, whereby MYB and green fluorescent protein (GFP) were co-expressed by using the pLV411G vector [37,38]. Successfully transduced cells were selected by cell sorting for GFP.

**Table 1:** Details of shRNAs used in current study

**Gene**	**Clone ID**
Zeb1sh var1	V2LHS_116662
Zeb1sh var2	V2LHS_116659
Zeb1sh var3	V2LHS_116663
Zeb1sh var4	V2LHS_226625
MYBsh var1	V2LHS_36797
MYBsh var2	V2LHS_36795
CDH1sh A	V2LHS_14834
CDH1sh B	V2LHS_14838
CDH1 shC	V2LHS_14837
CDH1 sh D	V2LHS_243170

Zeb1 shRNAs were purchased directly from Open Biosystems; MYB and E-cadherin shRNAs were provided by the Victorian Centre for Functional Genomics (VCFG), Peter MacCallum Cancer Centre.

### Immunofluorescence microscopy, multiplex tandem polymerase chain reaction (MT-PCR), and quantitative real-time PCR (QRT-PCR)

These were performed as previously described [39]. Details of antibodies and QRT-PCR primers used are provided in Tables 2 and 3, respectively. Gene-symbol abbreviations used in the current study are shown in Table 4.

**Table 2:** Antibodies used for IF, ICC, and IHC

**Primary antibody**	**Species**	**Source**
Snail1	Rat	Cell Signaling Technology, Beverly, MA, USA (cat. no. 4719)
Snail2	Rabbit	Abcam, Cambridge, UK (cat. no. ab27568)
Zeb1	Goat	Santa Cruz Biotechnology, Santa Cruz, CA, USA (cat. no. sc-10572)
Vimentin	Mouse	DAKO, Campbellfield, VIC (cat. no. H 7095)
E-cadherin	Rabbit	Abcam, Cambridge, UK (cat. no. ab40772)
E-cadherin	Mouse	BDT (cat. no. 612130)
MYB	Mouse	Millipore, Billerica, MA, USA (cat. no. 05–175)
α-pan actin	Mouse	Abcam, Cambridge, UK (cat. no. ab75373)
ER-α	Mouse	DAKO, Campbellfield, VIC (cat. no. M 7047)
Cytokeratin	Mouse	DAKO, Campbellfield, VIC (cat. no. M 0821)
CT3 *MYB* w CT5	Mouse	Synthesized in lab of Prof. Gonda
**Secondary antibody**	**Species**	**Source**
Anti-mouse Alexa 488	Goat	Invitrogen, Camarillo, CA, USA (cat. no. A-11011)
Anti-mouse biotin	Rabbit	DAKO, Campbellfield, VIC (cat. no. E0354)
Anti-goat biotin	Rabbit	Vector Laboratories, Burlingame, CA, USA (cat. no. BA-5000)
Anti-rabbit biotin	Swine	DAKO, Campbellfield, VIC (cat. no. E0431)
**Tertiary label**		**Source**
Streptavidin/HRP		DAKO, Campbellfield, VIC (cat. no. P0397)
DAPI		(4′,6-Diam idino-2-phenylindole, dihydrochloride)

**Table 3:** Primers used in QRT-PCR

**Gene**	**Forward primer sequence**	**Reverse primer sequence**
L32	GATCTTGATGCCCAACATTGGTTATG	GCACTTCCAGCTCCTTGACG
Snail1	CCAGACCCACTCAGATGTCAAGAA	GGCAGAGGACACAGAACCAGAAAA
Snail2	CCCAATGGCCTCTCTCCTCTTT	CATCGCAGTGCAGCTGCTTATGTTT
Twist1	CTAGAGACTCTGGAGCTGGATAACTAAAAA	CGACCTCTTGAGAATGCATGCATGAAAAA
Zeb1	GTTACCAGGGAGGAGCAGTGAAA	GACAGCAGTGTCTTGTTGTTGTAGAAA
MYB	CAGGAAGGTTATCTGCAGGAGTCTTCAAAA	CTATAGGCGGAGCCTGAGCAAAA
E-cadherin	GCCCTGCCAATCCCGATGAAA	GGGGTCAGTATCAGCCGCT
Vimentin	GCTTCAGAGAGAGGAAGCCGAAAA	CCGTGAGGTCAGGCTTGGAAA
ERα	GGAGCACCCAGGGAAGCTACTGTTT	GATCTCCACCATGCCCTCTACACATTTT
MKI67	GTAGGTGAGGGCAAAGGCACGAAA	CTTCCGCTTTGCAGGTTGCTTAAA
Primers for ChIP	AGCAGGTGGGAATTCGTTCC	CCAACGTCCGGATACATTTC

**Table 4:** Definition of gene symbols and names used in current study

**Gene symbol**	**Detailed name**
CA9	Carbonic anhydrase IX
CD24	CD24 molecule (Indian blood group)
CD44	CD24 molecule (Indian blood group)
CDH1	Cadherin 1, type 1, E-cadherin (epithelial)
CDH13	Cadherin 13, H-cadherin (heart)
CDH2	Cadherin 2, type 1, N-cadherin (neuronal)
CDH5	Cadherin 5, type 2 (vascular endothelium)
CTGF	Connective tissue growth factor
CYR61	Cysteine-rich, angiogenic inducer, 61
E12/E47	Transcription factor 3 (E2A immunoglobulin enhancer binding factors E12/E47)
EPCAM	Epithelial cell adhesion molecule
ESR1	Estrogen receptor 1
FOXC2	Forkhead box C2 (MFH-1, mesenchyme forkhead 1)
GSC	Goosecoid homeobox
KRT7	Keratin 7
MKI67	Antigen identified by monoclonal antibody Ki-67
MMP1	Matrix metallopeptidase 1 (interstitial collagenase)
MMP13	Matrix metallopeptidase 13 (collagenase 3)
MMP14	Matrix metallopeptidase 14 (membrane-inserted)
MMP2	Matrix metallopeptidase 2 (gelatinase A, 72-kDa gelatinase, 72-kDa type IV collagenase)
MMP9	Matrix metallopeptidase 9 (gelatinase B, 92-kDa gelatinase, 92-kDa type IV collagenase)
MYB	V-myb myeloblastosis viral oncogene homolog (avian)
PAX2	Paired box 2
PAX6	Paired box 6
PLAU	Plasminogen activator, urokinase
SNAI1	Snail homolog 1 (Drosophila)
SNAI2	Snail homolog 2 (Drosophila)
TWIST1	Twist homolog 1 (Drosophila)
VIM	Vimentin
ZEB1	Zinc-finger E-box binding homeobox 1
ZEB2	Zinc-finger E-box binding homeobox 2

Annotation according to the National Center for Biotechnology Information, U.S. National.

Library of Medicine online Gene search.

### Immunobead-negative selection and FACS analysis for EpCAM

LA, SCRsh-ET (SCR, scrambled control shRNA) and ZEB1sh-ET were trypsinized and counted, and approximately 10^6^ cells were added to 20 μl of beads (Collection Epithelial Enrich, Dynal (Invitrogen) to a final volume of 5 ml and incubated at 4°C with rotation for 2 hours. The bead-cell mixture was then placed in a DynaMag-15 magnet (Invitrogen) to separate beads from solution. The number of unbound cells was then determined and represented as a proportion of the original population. Anti-human EpCAM and CDH1 antibodies (Biolegend, San Diego, CA, USA) were used for fluorescence-activated cell sorting (FACS). Cells were trypsinized and counted, and approximately 10^6^ cells in PBS were incubated with 20 μl of antibody for 30 minutes in the dark at 4°C. Cells were then washed twice with 1 ml of phosphate-buffered saline (PBS) and resuspended in 300 μl of PBS, transferred to a 5-ml polystyrene tube and fluorescence measured by using the LSR Fortessa II (BD Biosciences). Data were analyzed by using FlowJo Software (version 7.6.4).

### BrdU (bromodeoxyuridine) pulse labeling and flow cytometry, sulforhodamine B (SRB) colorimetric assay, Western blotting

These were performed as previously described [40-43].

### Clonogenic assay and colony morphology analysis

**Figure 1:**
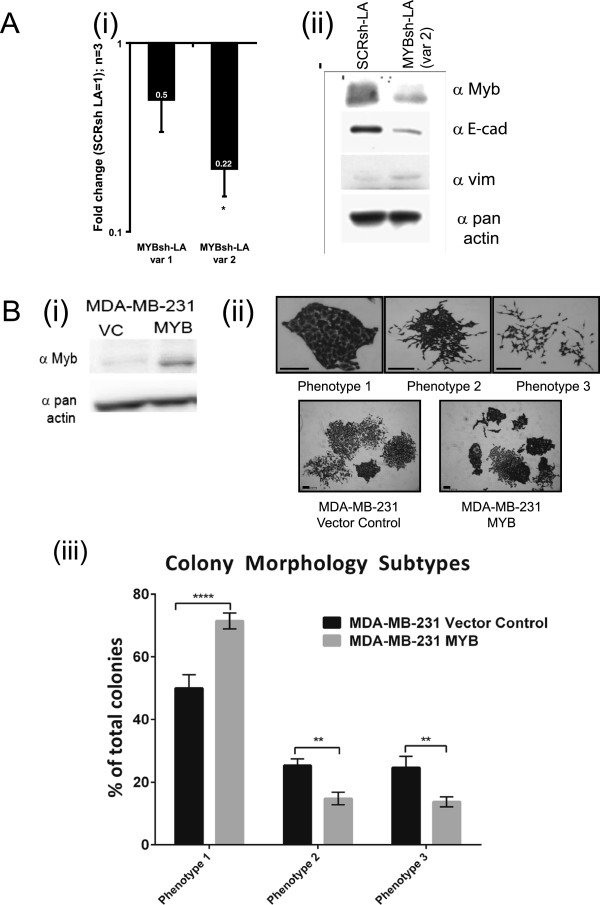
**Manipulation of MYB expression influences epithelial/mesenchymal gene expression and clonal morphology. (A)** Stable knockdown of MYB in LA cells by shRNA led to mesenchymal gene-expression changes **(i)** Analysis of MYB expression reduction (notation on graph bar indicates level of fold reduction; *n* = 3, error bars represent SEM, *, significance set at *P* < 0.05, Student paired *t* test) and **(ii)** further validation of MYB knockdown and analysis of EMT markers CDH1 and VIM with Western blot. **(B)** Colony morphology was significantly altered in MYB-overexpressing MDA-MB-231 cells. **(i)** MYB overexpression validated by Western blotting; **(ii)** examples of the three different colony phenotypes ranging from more-epithelial/compacted (phenotype 1) to most-mesenchymal/scattered (phenotype 3), and representative images of colonies seen in vector control- or MYB-transfected MDA-MB-231 cells, and **(iii)** increased epithelial (phenotype 1) and decreased mesenchymal (phenotypes 2, 3) seen after MYB transfection of MDA-MB-231 cells (*n* = 2; ANOVA. **P < 0.005; ****P < 0.0001).

Cells were trypsinized to a single-cell suspension, and 2,000 cells plated in 10 ml of media in an 8-cm-diameter culture dish. After 9 to 10 days, the cells were fixed in 10% neutral buffered formalin, stained with crystal violet (0.5% wt/vol), and washed with PBS. Plates were dried overnight face down. Images were taken of each plate by using the VersaDoc system, and a ‘‘thresholded” image was generated by the imaging program Image J when particular upper and lower pixel-intensity values were set (in the case of the images in Additional file 1: Figure S1C, and Additional file 2: Figure S2B, it was set to automatically detect the threshold, usually at pixel intensities between 129 and 255). This effectively segmented the image into colonies and background. Colonies were then counted by using this imaging program. Colony morphology was assessed by counting a minimum of 100 colonies/plate and classifying them based on the phenotypes displayed in Figure 1B(ii).

### Monolayer wound-healing assay

MYB-transfected MDA-MB-231 cells and sh-MYB-knocked-down LA cells were plated in a 24-well plate set up in triplicate and incubated at 37°C for 24 hours to allow the formation of a confluent monolayer. The cells were then wounded by using a P200 pipette tip. The wounded monolayers were washed with complete media to remove the detached cells. Images of the wound were taken at 0, 24, and 48 hours. Wound areas at each time point were analyzed and quantitated by using ImageJ software.

### Transwell-migration assay

Cells were trypsinized to form a single-cell suspension and resuspended into invasion medium (serum-free RPMI supplemented with 0.1% BSA). Cell counts and viability were estimated by using the Countess Automated Cell Counter (Invitrogen). Then 1 × 10^5^ cells in 100 μl of invasion medium were added into the upper compartment of each migration chamber (6.5-mm Transwell with 8.0-μm pore polycarbonate membrane insert, Corning). The lower chamber contained 600 μl of chemoattractant medium (RPMI supplemented with 10% FBS and 0.1% BSA). The plates were then incubated at 37°C for 4 hours. The membranes were then washed twice in 1× PBS, fixed in cold methanol for 2 minutes, stained with Diff-Quick (1 minute with eosin, followed by 1 minute with buffered thiazole), and washed with water to remove any excess stains. The nonmigrated cells that remained on the upper surface of the membrane were removed by carefully wiping with a cotton bud. The membranes were left to air dry and subsequently mounted on slides. Five random high-power fields per membrane were imaged and counted by using ImageJ software.

### Live cell-migration analyses

Live-cell imaging was performed on cells plated at 1 × 10^5^ cells/dish in 3-cm TC glass-bottom dishes (FluoroDish; World Precision Instruments, Inc., Sarasota, FL, USA) by using magnification × 10 phase-contrast objective on an Olympus IX70 microscope attached to a Spot Monochrome camera (2.1.1). Images were acquired for 120 to 150 frames at one frame per 2 minutes, by using Image-Pro Plus 4.5 (MediaCybernetics, Silver Spring, MD, USA). Analysis was via Image-Pro–Analyser 6.1 (MediaCybernetics).

### Immunocytochemistry

Antibodies are detailed in Table 2. Cells were cultured on Superfrost plus slides (Thermo Scientific, Scoresby, VIC, Australia) overnight then fixed in ice-cold methanol for 20 minutes at −20°C. After 3× PBS 5-minute washes, slides were immunostained by following the protocol described in the Immunohistochemistry section later.

### Human breast primary and matched sentinel lymph-node sections

Formalin-fixed, paraffin-embedded surgical specimens were obtained from St. Vincent’s Pathology, Melbourne, with approval of the St. Vincent’s Hospital Human Research Ethics Committee (006–09). Specific patient consent was not required for this study, on the basis that (i) the tissues were collected by the hospital for hospital procedures, (ii) individual patients could not be identified, and (iii) this project did not affect tissue donors’ disease or treatment.

### Immunohistochemistry

Antibodies are detailed in Table 2. Paraffin-embedded blocks were sectioned, mounted on Superfrost plus slides, de-waxed, and then brought to water. Slides were incubated in primary antibody overnight at 4°C (all antibodies were diluted in 0.1% bovine serum albumin (BSA)/PBS), washed in 0.1% Tween20/1 × PBS, and then incubated with a biotinylated secondary antibody. Slides were then washed in 1 × PBS and incubated with streptavidin-linked HRP tertiary antibody. Staining was visualized with peroxidase-sensitive Sigmafast 3,3′-diaminobenzidine tablets (DAB; Sigma), resuspended to 0.5 mg/ml in 50 m*M* Tris, pH 7.6, containing 150 μl H_2_O_2_. Slides were counterstained with Mayer’s hematoxylin solution (Amber Scientific, Midvale, WA) and mounted in DPX (BDH, Poole, England).

### Chromatin immunoprecipitation (ChIP) assay

SCRsh-ET and ZEB1sh-ET cells were grown in T175 flasks to 70% to 80% confluence (approximately 2 to × 10^7^ cells). Cells were washed 3 times with PBS and incubated 20 minutes at room temperature with 1% formaldehyde. Cross-linking was stopped by the addition of 0.1 *M* glycine for 5 minutes at room temperature. Cells were washed 3 times with PBS and resuspended in 1.0 ml of lysis buffer (50 m*M* Tris–HCl, pH 8.1, 1.0% SDS, 10.0 m*M* EDTA, and protease inhibitor cocktail). Chromatin was sonicated (Bioruptor UCD-200, Diagenode) for 10 cycles of 20-second sonication by using 40% efficiency with 2-minute incubation at 4°C between the sonication steps to achieve an average chromatin length of 1 kbp. Chromatin extracts were centrifuged for 14,000 rpm for 30 minutes at 4°C, and the supernatant was diluted 1:10 with IP dilution buffer (20 m*M* Tris–HCl, pH 8.1, 1.0% Triton X-100, 2.0 m*M* EDTA, 150 m*M* NaCl). Diluted chromatin extracts were pre-cleared with ProteinA/G-agarose (Santa Cruz) for 1 hour at 4°C and subsequently used for immunoprecipitation with 2 μg of ZEB1 antibody (E-20; Santa Cruz) or 2 μg of control anti-goat IgG antibody overnight at 4°C. Immunoprecipitants were collected by the addition of 100 μl ProteinA/G-agarose (Santa Cruz) precoated with BSA and salmon sperm DNA. After incubation for 1 hour at 4°C, the agarose was pelleted and washed for 15 minutes with each of the following buffers: low-salt buffer (20 m*M* Tris–HCl, pH 8.1, 0.1% SDS, 1.0% Triton X-100, 2.0 m*M* EDTA, and 150 m*M* NaCl), high-salt buffer (20 m*M* Tris–HCl, pH 8.1, 0.1% SDS, 1.0% Triton X-100, 2.0 m*M* EDTA, 500 m*M* NaCl), LiCl buffer (10 m*M* Tris HCl, pH 8.1, 1 m*M* EDTA, 1.0% deoxycholate, 1.0% NP-40, 0.25 *M* LiCl), followed by three washes in 1× TE. Complexes were eluted with 500 μl elution buffer (1.0% SDS, 0.1 *M* NaHCO3). Eluates were adjusted with NaCl (0.2 *M*) and cross-links reversed by heating the samples at 65°C overnight. Eluates were adjusted with EDTA (10 m*M*) and 50 m*M* Tris HCl, pH 6.5) and subsequently treated with Proteinase K (2 μl of 10 mg/ml) for 1 hour at 45°C. DNA was recovered by phenol/chloroform/isoamyl alcohol extraction and ethanol precipitation and resuspended in 50 μl 1× TE. Samples were quantified with SYBR-green real-time PCR analysis (Applied Biosystems). Details of primers used for Q-PCR analysis are found in Table 3. Results were expressed as the percentage of total DNA immunoprecipitated with ZEB1 antibody or control anti-goat IgG relative to unprecipitated input samples that were used as reference for all QRT-PCR reactions.

### Plasmid DNA, transfection, and reporter gene assay

**Figure 2:**
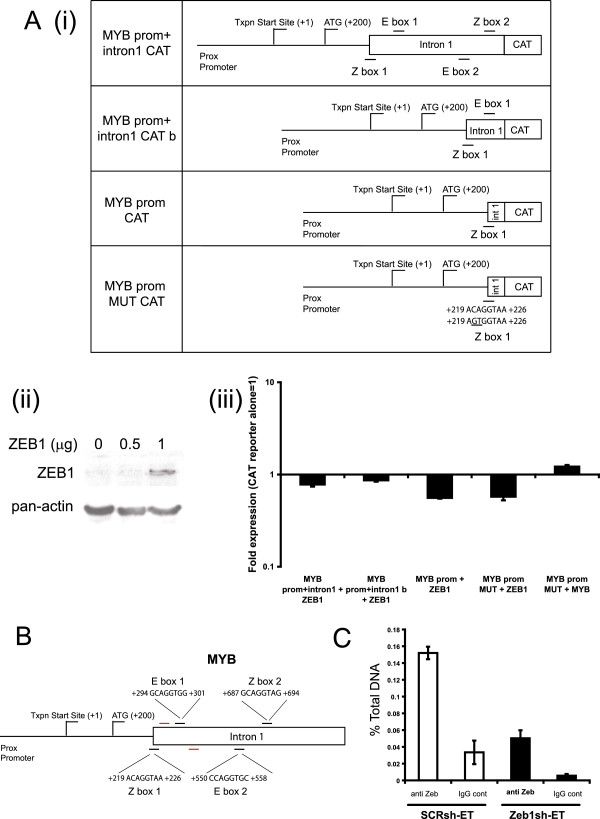
**Regulation of the MYB promoter by ZEB1. (A) (i)** Outline of several CAT reporter constructs containing *MYB* promoter and intron 1 sequences with various numbers of E and Z boxes, which were used in the CAT ELISA assay. **(ii)** Western blot of HEK 293 cells transfected with various amounts of pcDNA3.1-ZEB1 expression construct, as surrogate validation of ZEB1 expression in the CAT ELISA assay. **(iii)** Results of a CAT ELISA assay, “+ZEB1” expressed as fold change from each individual CAT reporter alone, *n* = 1; error bars represent standard deviation. **(B)** Scheme of the *MYB* gene indicating (black bars) the position of E-boxes and Z-boxes, sites at which ZEB1 may bind [4]. Red bars indicate QRT-PCR primers used in the ChIP assay. **(C)** ChIP analysis of SCRsh-ET and ZEB1sh-ET cells using anti-ZEB1 (E20; Santa Cruz) and control anti-goat IgG. The graph depicts the enrichment of PCR-amplified immunoprecipitated DNA expressed as a percentage of total DNA immunoprecipitated with ZEB1 antibody or control anti-goat IgG relative to unprecipitated input sample. Data are representative of three independent assays (error bars represent SD). Additional data are shown in Additional file 9: Figure S9B.

pMYB prom + intron1 CAT (chloramphenicol acetyltransferase) and pMYB prom CAT were described in a previous study [44], and pMYB prom + intron1 CAT b (used in this study) was in the previous study named 5′-3′. In pMYB prom + intron1 CAT construct, the promoter, exon 1, and intron 1 sequences of *MYB* gene were cloned upstream of the CAT. Both sets of E/Z boxes identified in Figure 2A were included in this construct. pMYB prom + intron1 CAT construct b was a shortened version, containing only the first set of Z/E boxes upstream of CAT. pMYB prom contained the promoter region only, encompassing only the first Z-box. This was then mutated by using the QuickChange Site-Directed Mutagenesis Kit (Stratagene/Integrated Sciences), such that CAGGTA was mutated to *GT*GGTA, and named MYB prom MUT CAT. Expression of exogenous ZEB1 from the expression construct (pcDNA3.1-ZEB1) [45] was confirmed with Western blot by using a goat anti-ZEB1 antibody C-20, sc-10570; Santa Cruz). A β-galactosidase expression vector was used as an internal control in the reporter assay. Approximately 7 μg of total plasmid DNA was transfected into HEK293T cells by using Fugene HD. In brief, 1.6 × 10^6^ cells/T25 tissue-culture flask was seeded, and transfections were carried out according to manufacturer protocol. After 72 hours of transfection, cells were lyzed by using 1 ml of lysis buffer/T25 flask, and 200 μl of extract was used to quantitate CAT reporter gene activity by using a CAT-ELISA enzyme immunoassay kit (Roche Applied Science, Castle Hill, NSW, Australia).

### Statistical analyses

Student paired *t* tests were performed by using Microsoft Excel version 2003 (18.8335.8333) SP3. Mann–Whitney and analysis of variance (ANOVA) tests were performed by using Graph Pad Prism (GraphPad Software, San Diego, CA, USA). Where “n” is used (as in *n* = 3), this refers to the number of independent, biologic replicate experiments performed.

## Results

### Gene-expression analysis in the PMC42 EMT model

**Figure 3:**
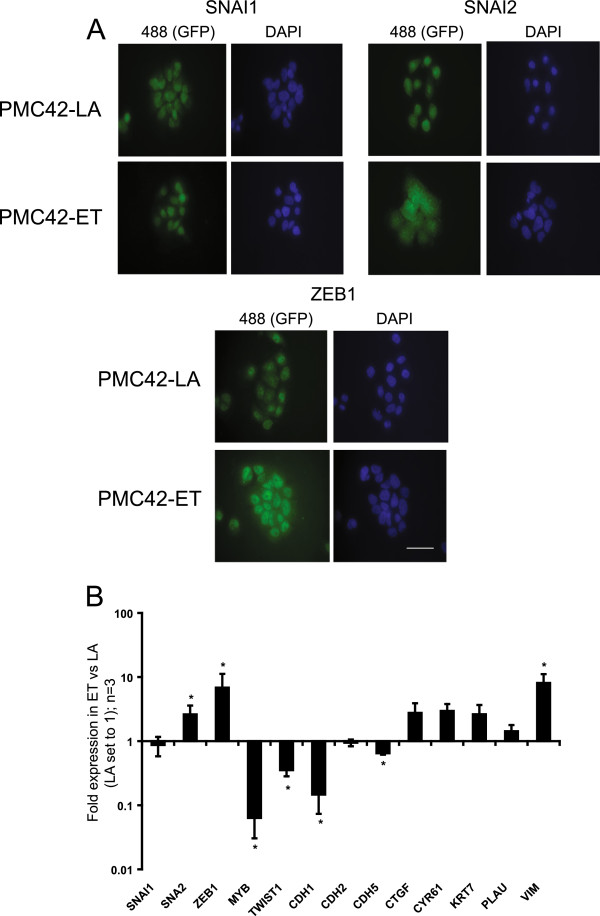
**MET/EMT cell states in the LA/ET breast cancer cell system. (A)** Immunofluorescence for CDH1-repressor genes SNAI1, SNAI2, and ZEB1 (as indicated with green fluorescence (488) and nuclei stained with 4′,6-diamidino-2-phenylindole (DAPI), magnification 600×, scale bar, 50 μm; and **(B)** their expression along with other EMT-related genes, and MYB. Fold gene expression (for all QRT-PCR in this article, this is first corrected to housekeeping ribosomal protein L32; MYB, SNAI1, SNAI2, and ZEB1 are QRT-PCR data; all others are MT-PCR first corrected to the housekeeping genes L32, GUSB, and NONO) is shown, relative to PMC42-LA, which has been set to 1. Data shown are an average of three independent biologic replicates, error bars represent SEM. Student paired *t* test was used to determine significance; *P* < 0.05.

The parental ET cells display mesenchymal characteristics compared with the more-epithelial LA derivative, providing a unique opportunity to investigate the epithelial –mesenchymal axis in isogenic breast cancer cell lines [6,31,32]. In investigating the cellular localization and expression of three significant EMT regulators, SNAI1, SNAI2, and ZEB1 [5] between LA and ET cells, ZEB1 nuclear staining and mRNA abundance had the greatest differential and favored the ET cells (Figure 3A, B). By contrast, MYB expression was significantly higher in LA cells (Figure 3B). ET cells also expressed lower levels of E-cadherin (CDH1) and higher levels of vimentin (VIM) than did LA cells (Figure 3B), as previously shown immunocytochemically [32]. Somewhat surprisingly, lower levels of Twist1 (TWIST1) were seen in ET cells compared with LA (Figure 3B).

### ZEB1sh-ET cells are epithelially shifted, express higher MYB, and are more proliferative

**Figure 4:**
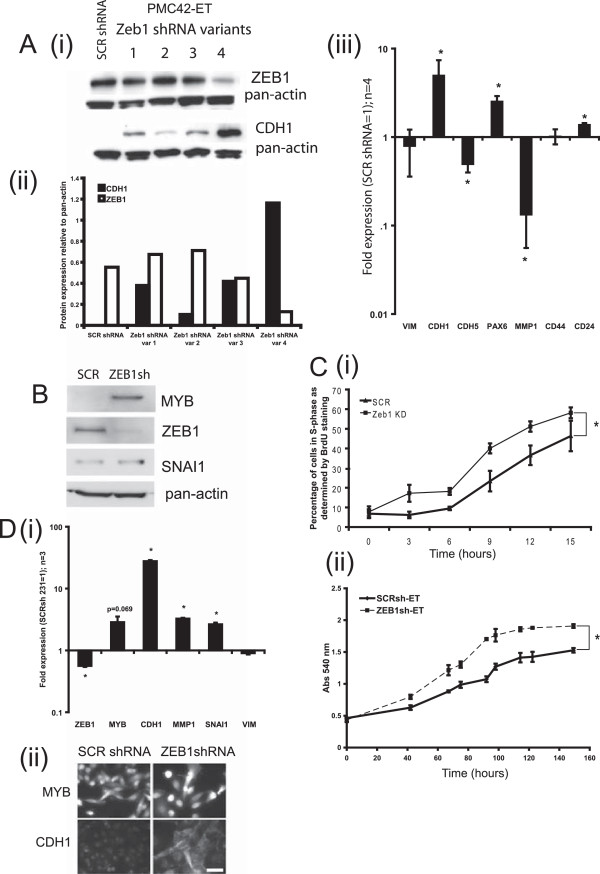
**ZEB1sh-ET cells are more epithelial, express higher MYB, and are more proliferative than SCRsh-ET controls. (A) (i)** Western blotting for ZEB1 and CDH1 in PMC42-ET cells transfected with shRNA variants 1 to 4; **(ii)** bar graph of band intensity of the Western blot shown in **(i)**. **(iii)** Expression (MT-PCR) of EMT-related genes, ZEB1sh-ET relative to SCRsh-ET; fold expression shown. Data shown are the average of four independent biologic replicates; Student paired *t* test was used to determine significance (*), set at *P* < 0.05; error bars represent SEM. The expression of other EMT-related genes not shown in this figure is shown in Additional file 1, part A. **(B)** ZEB1sh-ET express higher MYB protein and mRNA (Additional file 3D) and are more proliferative **(C)**. **(i)** Percentage of ZEB1sh-ET cells in *S* phase as determined by BrdU staining after release from mitotic arrest with nocodazole; **(ii)** Growth rate as shown by the SRB growth assay. Results shown (**C**, part i) are from three independent experiments; error bars represent SEM, and the repeated-measures two-way ANOVA statistical test was used to determine significance, *P < 0.05. Result shown in **(ii)** is one representative SRB assay, of a total of three, all of which were found to be statistically significant by the repeated-measures two-way ANOVA statistical test. Further replicates are found in Additional file 1B. **(D)** ZEB1 knockdown in MDA-MB-231 breast cancer cells: **(i)** characterization of ZEB1 knockdown (shRNA) MDA-MB-231 cells by QRT-PCR; **(ii)** immunocytochemistry showing that ZEB1 knockdown results in reexpression of E-cadherin at the cell membrane, and MYB nuclear reexpression; magnification, 400×, scale bar, 50 μm.

We previously showed that transient knockdown of ZEB1 with siRNA caused re-expression of CDH1 in ET cells [32]. Here we established stable ZEB1 knocked-down ET cell lines with four shRNA variants and a”:scrambled” nontargeting hairpin control (SCRsh-ET). The level of ZEB1 knockdown was proportional to CDH1 reexpression (Figure 4A, parts i, ii; Further characterization of ZEB1sh-ET cells is shown in Additional file 3). Variant number 4 (hereafter designated ZEB1sh-ET) displayed the greatest ZEB1 knockdown and commensurate CDH1 protein reexpression. Examination of the expression of EMT-related genes revealed that ZEB1 knockdown in ET cells caused MET-like gene-expression changes, including increased expression of CDH1 and CD24, and reduced expression of MMP1, a known target of ZEB1 for induction [46] (Figure 4A, part iii). Further proepithelial changes due to ZEB1 knockdown are shown in Additional files 3 and 4. These experiments suggested that suppression of ZEB1 caused gene-expression changes similar to the paired LA-versus-ET model of MET. Similarly, expression of MYB protein (Figure 4B) and mRNA (Additional file 3D) was higher in ZEB1sh-ET cells. Pax-2 was also increased (Figure 4A, part iii), but no change was seen in a variety of other markers, including CDH4, CDH13, ZEB2, SNAI1, SNAI2, TWIST1, MMP9, MMP13, and MMP14 (Additional file 1A).

ZEB1 knockdown in ET cells was associated with an increased rate of proliferation, as shown by BrdU (bromodeoxyuridine) pulse labeling and flow cytometry (Figure 4C, part i). Over the 15-hour period after arrest, we observed a significantly higher proportion of ZEB1sh-ET cells in *S* phase compared with SCRsh-ET cells, indicative of an increase in proliferation. The ZEB1sh-ET cells also proliferated faster than SCRsh-ET cells in the sulforodamine B (SRB) growth assay [47], (Figure 4C, part ii, further repeats shown in Additional file 1B). ZEB1sh-ET cells also grew larger colonies from a single cell compared with the SCRsh-ET cells (Additional file 1C), an increase that was negated when MYB was knocked down (Additional file 2A and B). Fewer colonies were seen after ZEB1 knockdown, suggesting that ZEB1 may control other models of regulation including survival, apoptosis, anoikis, all of which have been linked to EMT. In MDA-MB-231 cells, ZEB1 knockdown also led to increased CDH1 at the cell membrane and MYB expression (Figure 4D, part i), the latter being shown to be nuclear (Figure 4D, part ii). Consistent with previous work [10], we observed that these ZEB1-knockdown cells, when plated in 3D, exhibited diminished sprouting and retarded wound closure (data not shown).

### MYB and ZEB1 expression are inversely correlated in various *in vivo* and *in vitro* biologic settings

**Figure 5:**
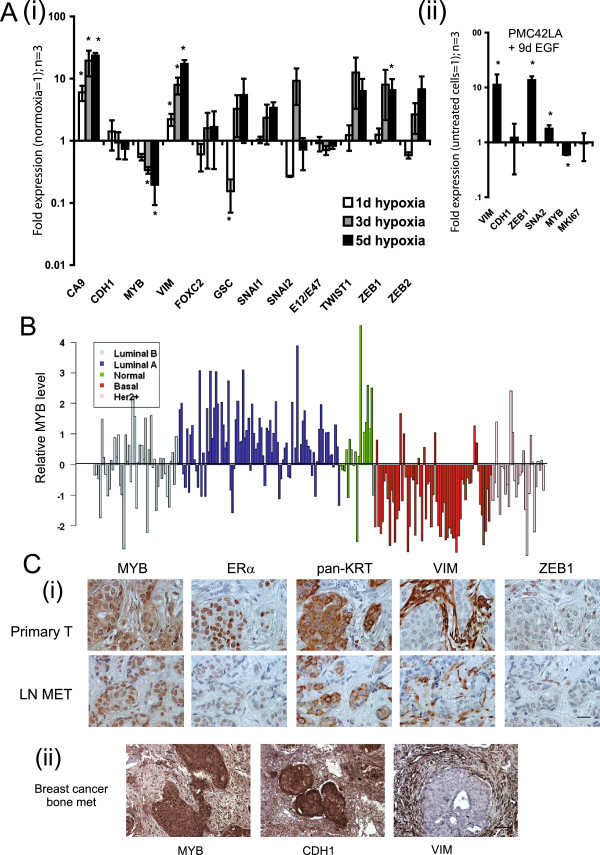
**MYB and ZEB1 expression are inversely correlated in various *in vivo *and *in vitro *biologic settings. (A)** MYB expression is reduced in EMT. **(i)** Exposure of MDA-MB-468 cells to hypoxia (1% oxygen) for up to 5 days led to significant (*P < 0.05, repeated measures one-way ANOVA with Dunnet multiple comparison test) induction of the hypoxia-indicator gene carbonic anhydrase 9 (CAIX) and significant EMT-related gene changes, including the induction of ZEB1 and repression of MYB. **(ii)** Treatment of PMC42-LA cells with 10 ng/ml EGF for 9 days (EGF-containing media replenished every 3 days) induced an EMT in which ZEB1 expression was significantly (*P < 0.05, Student paired *t* test) induced and MYB repressed. For both **(i)** and **(ii)**, results shown are QRT-PCR data, expressed as fold change (corrected to untreated cells), of three independent experiments, and error bars represent SEM. Morphologic changes of MDA-MB-468 cells exposed to hypoxia and EGF-treated PMC42-LA are found in Additional file 5A and B, respectively. Further evidence of MYB-expression reduction in EMT is found in Additional file 6A and B. **(B)** Plot of expression of MYB across various breast tumors of increasing aggressiveness, using data derived from a publically available microarray dataset [50]. **(C)** MYB tumoral staining correlates with CDH1 and inversely correlates with VIM in **(i)** matched human breast tumor primary (Primary T) and sentinel lymph node metastases (LN MET); and in **(ii)** nonrelated human breast cancer bone metastases. Magnification 400×; scale bar, 150 μm. Findings further supported by IHC shown in Additional file 5D.

We examined the ZEB1/MYB relation more broadly in EMT and in human breast tumors. Hypoxia has been reported to induce EMT in MDA-MB-468 cells [48,49]. In a time course of 5 days of hypoxia, only ZEB1 was significantly induced of the eight CDH1 repressor genes examined (Figure 5A, part i, immunocytochemistry for E-cadherin and vimentin: Additional file 5A). This was observed at day 5, when MYB expression was also significantly reduced. MYB repression also accompanied hypoxia-induced EMT-like expression changes in MCF-7 cells (see Additional file 6A).

After 9-day treatment of PMC42-LA cells with 10 ng/ml EGF, VIM and ZEB1 expression were upregulated, and MYB expression was downregulated (Figure 5A, part ii, morphologic changes: Additional file 5B). EGF-induced EMT also led to the repression of MYB in the cell line T47D (Additional file 6B).

Given that tumor aggressiveness has been linked with EMT ([51], reviewed in [52]) we examined the expression of MYB in a publically available microarray dataset series of human breast tumors [50] and observed that MYB expression was generally high in *Luminal A* and *Normal-like* tumors but dramatically lower in *Basal* and *Her2 +* tumors (Figure 5B). This finding is consistent with previous work that also showed that MYB expression in luminal tumors correlated with a good prognosis [53]. Analysis of publically available expression data [13] from 51 human breast cancer cell lines that clustered into Luminal, BasalA, and BasalB subgroups [6,11] also revealed significantly higher MYB expression in Luminal and Basal A subgroups compared with Basal B (Additional file 5C). This pattern was the inverse to that of ZEB1, but correlated with CDH1.

In our study, serial sections from paraffin-embedded matched tissue sets of primary tumors and involved lymph nodes from 11 individual patients were immunostained with the various markers listed in Figure 5C, part i. Histologic examination of another primary tumor-lymph node set from this group of 11 is shown in Additional file 5D. MYB was found to be nuclear and generally tumoral, and associated with epithelial markers CDH1 and cytokeratin along with ERα, particularly in primary tumor samples (invasive ductal breast carcinomas). MYB and associated markers were inversely expressed with regard to VIM. This is further supported by a positive correlation of MYB and CDH1 in breast cancer bone metastases, with both markers staining inversely to VIM (Figure 5C, part ii).

### The manipulation of MYB expression influences epithelial versus mesenchymal state

Given that ZEB1 knockdown led to MYB reexpression and epithelialization, we investigated whether these changes were causally linked. Knockdown of MYB mRNA (Figure 1A, i) and protein (Figure 1A, ii) in the more-epithelial LA cells (MYBsh-LA) reduced CDH1 and induced VIM protein expression (Figure 1A, ii). To investigate this further, we created a stable MYB-overexpressing MDA-MB-231 cell line (Figure 1B, part i), and found a shift from mesenchymal, scattered colonies to more-compact epithelial colonies in these cells compared with vector control cells, when plated very sparsely, such that colonies would be seeded from single cells (Figure 1B, part ii). Despite these morphologic changes, preliminary experiments showed no effect of MYB manipulation on monolayer wound closure in either the MYB-transfected MDA-MB-231 cells or sh-MYB-knocked-down LA cells (*n* = 1; data not shown), and although a trend toward reduced Transwell migration was seen after MYB transfection of MDA-MB-231 cells, this was not significant (*n* = 3; data not shown). However, live cell-imaging analysis of monolayer cells plated relatively sparsely revealed an altered morphology and migration in the MYB-transfected MDA-MB-231 cells, which grew mostly as connected, flatter cells in epithelial groups compared with a carpet of smaller, fibroblast-like, bipolar, and round cells in the control cultures, and showed a slower, more-tortuous migration pattern than the vector control cells over a 4-hour period (see Additional file 7). In the 9-day EGF-EMT treatment regimen used previously, MYB knockdown appeared further to enhance the repression of CDH1 (see Additional file 8A, *n* = 1). In MYB-overexpressing MCF-7 cells, the epithelial phenotype was enhanced, as evidenced by significant reductions in VIM and SNAI1 expression (Additional file 8B).

### Potential reciprocal repression of ZEB1 by MYB

**Figure 6:**
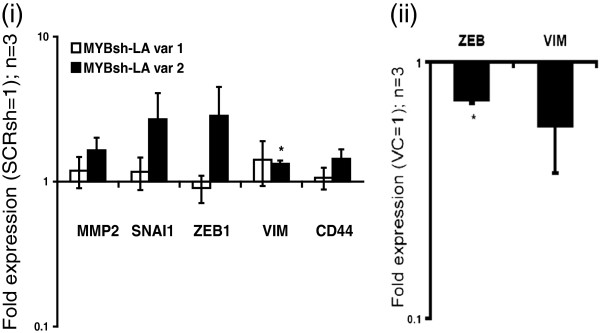
**Potential reciprocal repression of ZEB1 by MYB. (i)** A trend for ZEB1 upregulation was observed in MT-PCR analysis of mesenchymal gene expression in MYBsh-LA variants 1 and 2. **(ii)** ZEB1 was significantly downregulated in MYB-overexpressing MDA-MB-231. QRT-PCR expression analyses showing fold change (vector control set to 1); *n* = 3, error bars represent SEM. Significance is indicated by the asterisk, where *P* < 0.05. (Student paired *t* test used).

QRT-PCR analyses of the MYBsh-LA cells revealed an upregulation of ZEB1 expression in variant 2 (Figure 6(i)), the variant with the best MYB knockdown (Figure 1A, part i), suggesting that MYB represses ZEB1 expression in PMC42-LA. Similarly, ZEB1 was significantly repressed in MYB-transfected MDA-MB-231 cells (Figure 6(ii)), despite no detectable change in CDH1 expression (data not shown).

### MYB is a direct ZEB1 target

These experiments indicated that MYB and ZEB1 are inversely expressed in LA versus ET cells and in MDA-MB-231. We therefore reasoned that ZEB1 might directly repress MYB by binding to Z- or E-box consensus sequences within *MYB*. We identified four sequences within MYB that conformed to either the Z- or E-box consensus [4,5] (indicated as Z-box 1, E-box 1, E-box 2, and Z-box 2; Figure 2A part i and Figure 2B). Z-box 1 (ACAGGTAA) encompassed nt position +219 to +266, such that this sequence spanned the MYB exon 1 and intron 1 junction (Figure 2A, part I, and Figure 2B). E-box 1 (GCAGGTGG) encompassed nt position +294 to +301 and was positioned within intron 1 (Figure 2A, part I, and Figure 2B). E-box 2 (CCAGGTGC) encompassed nt position +550 to +558 and was positioned within intron 1 (Figure 2A, part I, and Figure 2B). Z-box 2 (GCAGGTAG) encompassed nt position +687 to +694 and was also positioned within intron 1 (Figure 2A, part i, and Figure 2B). Each of the Z- and E-box sites was conserved between human, mouse, and dog DNA sequences (Additional file 9A).

We next examined via a CAT reporter-gene assay whether ZEB1 has a direct functional effect on the MYB via the region containing Z-box 1, E-box 1, E-box 2 and Z-box 2 (Figure 2A, parts i and iii) . In this assay, we co-transfected an *MYB* promoter-intron 1-CAT construct containing Z-box 1, E-box 1, E-box 2, and Z-box 2 with a ZEB1 expression vector (pcDNA3.1-ZEB1) into HEK 293 cells and assessed CAT reporter activity (Figure 2A, parts i and iii). Protein expression from the ZEB1 construct was confirmed by Western blot (Figure 2A, part ii). CAT reporter-gene assays showed that the expression of exogenous ZEB1 in HEK 293 cells repressed the activity of the *MYB* promoter-intron 1-CAT reporter (Figure 2A, part iii). These data suggest that ZEB1 can negatively regulate *MYB*. This finding is consistent with the previous observations that have shown that ZEB1 acts as a repressor of gene activity [8-10].

To determine whether the Z-box 1, E-box 1, E-box 2, and Z-box 2 sequences contributed to the ZEB1-mediated repression of CAT activity, we next assessed a series of CAT-reporter constructs in which the Z- and E-box sequences were removed or mutated (MYB prom + intron 1 CATb, deletes E-box 2 and Z-box 2; MYB prom CAT, deletes E-box 1; MYB prom MUT CAT, mutates Z-box 1). CAT reporter-gene assays showed that ZEB1 repressed the activity of all of the deletion and mutant CAT reporter constructs including MYB prom MUT CAT (Figure 2A, parts i and iii). The MYB gene can positively autoregulate itself via sites in its proximal promoter [54]. Co-transfection of an MYB expression vector with MYB prom MUT CAT confirmed the activity of this reporter.

Collectively these data suggest that, in the context of the CAT reporter construct, the repressive activity of ZEB1 in HEK 293 cells was not dependent on the presence of the Z-box 1, E-box 1, E-box 2, or Z-box 2. We did not observe additional Z- or E-box consensus sites within the MYB promoter, exon1, or intron 1. This suggests that ZEB regulation of MYB may occur via ZEB1 binding to alternative nonconsensus site(s) within intron 1 or via alternative upstream sequences within exon 1 or the MYB promoter.

We next examined whether endogenous ZEB1 directly bound MYB *in vivo* by chromatin immunoprecipitation (ChIP) analysis. We undertook ChIP of SCRsh-ET and ZEB1sh-ET cells with an antibody directed against ZEB1, and experiments were performed at a standard chromatin fragment resolution of 1 kbp (see Methods). QPCR analysis of the immunoprecipitated chromatin used an optimized primer pair located at the 5′ end of intron 1 (Figure 2B). This approach allowed the analysis of ZEB1 occupancy of MYB across the region encompassing the 5′ end of intron 1 and the downstream proximal promoter and exon 1 (Figure 2B). ChIP data in Figure 2C suggest that in SCRsh-ET cells, MYB was enriched for ZEB1 binding. In contrast in ZEB1sh-ET cells, in which ZEB protein expression was decreased (shown by Western blot in Figure 4A, part i), ZEB1 binding was reduced (Figure 2C). Additional repeats of this experiment are shown in Additional file 9B. These data suggest that endogenous ZEB1 directly bound MYB *in vivo* in a region encompassing the 5′ end of intron 1 and the flanking proximal promoter and exon 1.

Collectively, the CAT reporter and ChIP analyses suggest that MYB is a target of ZEB1 and that ZEB1 exerts its repressive activity via nonconsensus sites located within the region spanning the 5′ end of intron 1 and the flanking proximal promoter of MYB.

## Discussion

**Figure 7:**
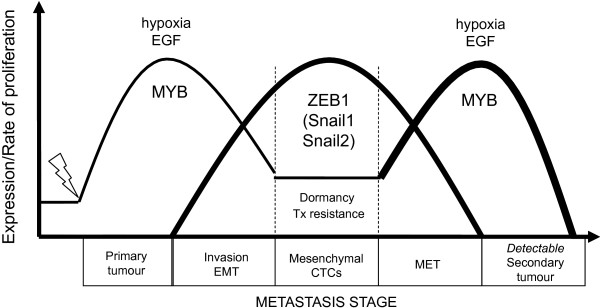
**Schematic depiction of the regulation of MYB expression, and hence proliferative rate (represented on the Y-axis) by ZEB1 at various metastatic stages (detailed on the X-axis).** Hypoxia/EGF positioned at the apex of each MYB curve indicates the switching on of EMT signals by these factors. This may lead to the induction of a cascade of CDH1-repressor genes, resulting in ZEB1 induction, which acts to stabilize the mesenchymal phenotype, resulting in further protection from immune-derived apoptotic signals within the bloodstream. Thus, these clinically indolent tumor cells may circulate and/or remain disseminated until a suitable, perhaps normoxic, secondary niche is found. Combined with other factors, such as hormone fluctuations associated with age and/or pregnancy or inflammation, a more conducive tumor microenvironment is created, triggering the reexpression of MYB, which may in turn repress ZEB1. The net result is the reversion of EMT (MET), and the secondary tumor actively grows until the cycle is repeated.

Breast cancer metastases in soft tissue and bone have been reported to reexpress CDH1 [55,56]. We have proposed that the restoration of proliferation required for a secondary tumor to form may be linked to the reversion of EMT, or MET, and we suggest that MYB plays a central role here in promoting tumor growth and the epithelial phenotype. Furthermore, we have established a functional and reciprocal relation between ZEB1 and MYB that is prevalent in breast cancer systems. Direct repression of MYB by the EMT driver ZEB1 builds onto the paradigm through which tumor growth, invasion, and metastasis are integrated (Figure 7). Resistance to apoptosis [57], reduced proliferation [5,58], and tumor dormancy [59,60] have all been associated with EMT in the past, but the potential role of MYB in this process is novel.

Examining the consequences of MYB knockdown in LA cells (Figure 1A), and the overexpression of MYB expression in MDA-MB-231 cells (Figure 1B) and MCF-7 cells (see Additional file 8B) has shown (a) that the manipulation of MYB expression has a direct consequence on epithelial versus mesenchymal state, and (b) MYB expression is concordant with the epithelial phenotype in terms of marker expression, culture and colony morphology, and random migration in the monolayer. We have also found increased MYB expression on CDH1 knockdown in MDA-MB-468 cells (not shown).

How could MYB expression determine epithelial state? One possibility that we have considered is through promotion of KLF4 expression. KLF4 was noted as a novel gene upregulated by MYB via occupation of the KLF4 promoter in MCF-7 cells treated with estradiol [61]. KLF4 is a metastasis-suppressor gene that drives CDH1 expression, preventing EMT [62]. Thus in an actively growing, ER-positive primary tumor, MYB may transduce estrogen signaling into both proliferation [16] and promotion of the epithelial phenotype. Further studies will be needed to determine whether this MYB-KLF4 partnership occurs in secondary tumors, whether this may be a means through which MYB could mediate the restoration of CDH1 at these sites, and the functional importance of the epithelial aspect regulated by MYB.

We present evidence to suggest that MYB may repress ZEB1 and hence relieve ZEB1-mediated CDH1 transcriptional repression, restoring the epithelial phenotype. This is suggested by the upregulation of ZEB1 on the knockdown of MYB in LA cells (Figure 6(i)) and the repression of ZEB1 in MYB-overexpressing MDA-MB-231 cells (Figure 6(ii)), and also by previous work in hematopoietic cells [19]. A predicted MYB binding site was identified in intron 1 of ZEB1, the site which is mutated in mice with multiple developmental malformations, some of which were suggested to be related to dysfunctional EMT or MET [20]. Thus, similar to the ZEB1-miR200 reciprocal regulatory relation [4], our data suggest a mutually antagonistic negative-feedback loop between ZEB1 and MYB. ZEB1 and MYB may therefore act as biologic switches, translating molecular changes in the tumor microenvironment into alterations in cell state. These may be epithelial or mesenchymal, as illustrated in Figure 7.

We have shown that ZEB1 binds to the MYB gene in a region encompassing the promoter and intron 1 (Figure 2B). Furthermore, CAT-reporter experiments suggest that the overexpression of ZEB1 represses MYB promoter-intron 1 activity (Figure 2C). This finding is supported by ET-LA cell comparative studies (Figure 3), the characterization of ZEB1sh-ET cells (Figure 4), and a potential role in the EMT of MDA-MB-468 (hypoxia) and LA cells (EGF) (Figure 5A), leading us to conclude that ZEB1 directly targets and represses MYB. Our deletion-mapping studies suggest that ZEB1 can operate through alternative regions, such as the MYB promoter. Although we did not identify exact consensus binding sites for ZEB1 within the promoter, this suggests that ZEB1 may work through divergent binding sites or indirectly by impinging on the activity of a transactivator, such as MYB. Given that MYB is a gene that positively autoregulates itself via tandem MYB binding sites located in the proximal promoter region [54], and that ZEB1 has been shown to inhibit the transcriptional activity of MYB [19], we propose that ZEB1 represses MYB gene expression by intercepting the positive-feedback cycle of MYB. ZEB1 downregulates miR34a in a similar manner by directly repressing the miR34a-positive transactivating protein, ΔNp63, resulting in the acquisition of invasive tumor cell capabilities [63].

Likewise, our studies outline the consequences of ZEB1-mediated gene repression, that is, the suppression of epithelial gene expression and cellular proliferation in breast cancer cells.

The role of MYB in EMT is controversial, and most likely is context dependent. MYB has been positively implicated in the induction of an EMT of avian embryonic neural crest cells [64] and recently in the induction of Snail2 expression in EMT of embryonic kidney, colon carcinoma, chronic myeloid leukemia-blast crisis, and neuroblastoma cells [65]. Knopfova *et al.*[66] recently showed increased migration and Matrigel invasion, but not collagen invasion, in MYB-transfected-MDA-MB-231 cells, which contrasts our live-cell imaging results for random monolayer migration. In further contrast to our results, it has been shown that TGF-β-induced EMT of ER-positive breast cancer cells is dependent on MYB upregulation, in which putative miR-200 family binding sites were identified in the MYB mRNA, and the downregulation of these miRs was pinpointed as the mechanism driving this EMT [67]. We have observed a significantly higher level of expression of miR-200 family in LA cells compared with ET (unpublished observation), but increased MYB expression in the LA cells that overexpress all miR-200 family members. In addition, in MYB-overexpressing MCF-7 or MDA-MB-231 cells, we did not observe an induction of Snail2 (Additional file 8B, C) nor did we find any association between MYB induction and EMT, but found the opposite (Figure 5A; Additional file 6A, B). We also found no association between MYB and Snail2 in Luminal versus Basal breast cancer subtypes (see Additional file 10). These dramatic differences could be due to cellular context, because much of our work was performed in the PMC42 model system, which clusters with Basal A/Basal breast cancer cell lines (T. Blick, E. Tomascovic-Crook, unpublished data). However, our results were supported by similar findings in luminal, ER^+^ breast cancer cells such as MCF-7 and T47D. It seems more likely that our findings are specific to ZEB1, which we find to be expressed later during the EMT, after SNAI1 and 2 induction in the EGF and EGF/staurosporine-induced EMT in our system [39]. Of the CDH1 repressor genes, ZEB1 has been found by others to be the more-dominant EMT driver, important in stabilizing and maintaining the mesenchymal phenotype in breast cancer systems [5,9,68]. It is possible that MYB contributes to early EMT responses by driving Snail 1 and 2 expression while repressing ZEB1, until stronger driving forces activate ZEB1, suppress MYB, and thus suppress SNAI1 and 2. Further work is required to delineate these potentially distinct EMT states and the capricious role that MYB may play.

Comparison of the EMT gene-expression profiles in control versus ZEB1 knockdown PMC42-ET cells (Figure 4A, part iii) may provide insights into specific gene-expression changes that may occur during MET at the secondary tumor site. For example, PAX6 was significantly upregulated in ZEB1sh-ET cells. PAX6 has been described as a tumor suppressor, suppressing invasiveness of glioblastoma cells and MMP2 expression [69], both of which are hallmarks of EMT [6]. PAX6 has also been implicated in promoting the proliferation of breast cancer cells [70], and we have reported that PAX6 is a direct MYB target gene in the neurogenic zones of the brain [71]. Thus the upregulation of PAX6 may also have contributed to the enhanced proliferative rate observed for ZEB1sh-ET. CDH5 expression was significantly downregulated in ZEB1sh-ET cells (Figure 4A, iii). CDH5 has been shown to be induced in an EMT of mammary tumor cells, influencing the levels of Smad2 phosphorylation and upregulating the expression of TGF-β target genes [72]. CD24 was found to be significantly increased in the ZEB1sh-ET cells, correlating with increased CD24 receptor expression, as determined by FACS (data not shown), consistent with our previous findings that EMT traits in human breast cancer cell lines correlate with the CD44(hi/+)/CD24(lo/-) stem-cell signature [12,73]. MMP1 expression was significantly reduced in the ZEB1sh-ET cells. Therefore, the ZEB1sh-ET cells reexpress markers of the epithelial phenotype.

## Conclusions

We have shown that ZEB1 transcriptionally represses MYB to achieve two outcomes: to downregulate cell proliferation and to stabilize the mesenchymal phenotype. Both outcomes are likely to be beneficial to the survival of the cancer cell in the systemic circulation and at sites of dissemination. EMT has been linked to the acquisition of cell dormancy and reduced chemotherapeutic efficiency (reviewed in [74]). In addition, a sustained mesenchymal state may enable escape from immune-related apoptotic signals via expression of factors such as SNAI1, which may act to suppress these signals and the immune system itself [75]; reviewed in [76]. An ongoing debate in relation to the treatment of metastatic breast cancer is whether to customize therapies that reactivate proliferation in dormant, circulating tumor cells and hence improve chemotherapeutic efficiency, or to maintain dormancy in these rogue cells [77,78]. Each approach is equally problematic, but gene-targeted modulation along the ZEB1-MYB axis may provide an avenue to tackle this foremost clinical dilemma.

### Note added in proof

As we noted earlier, the Raschella group initially reported that MYB expression was upregulated in ER-positive human breast cancer cell lines after TGF-β treatment because of transcriptional activation, release of miR-200 family suppression, and protein stabilization, and was instrumental in promoting EMT effects [67]. Since our manuscript was submitted, the Raschella group published a follow-up article [79] showing that MYB also activates the expression of the miR-200 family, which blocks ZEB1 expression. These new findings now support our key conclusion, that MYB favors the epithelial rather than mesenchymal state and opposes EMT. The authors also support our proposal of contextual issues surrounding MYB regulation of EMT processes in response to signals such as TGF-β, because in the combined presence of MYB and ZEB1, the ZEB1 suppression of miR200 is dominant. This may explain the discrepancies seen in our PMC42 model system compared with their original study. MYB seems poised for only transient expression in the epithelial setting because of the MYB-miR-200 feedback loop. The effects of TGF-β are also known to be very much context dependent.

## Abbreviations

2D: Two dimensional; 3D: Three dimensional; ANOVA: Analysis of variance; ATCC: American Type Culture Collection; bp: Base pair; BrdU: Bromodeoxyuridine; BSA: Bovine serum albumin; CAT: Chloramphenicol acetyltransferase; ChIP: Chromatin immunoprecipitation; DAB: 3,3′-diaminobenzidine; DMEM: Dulbecco modified eagle medium; EGF: Epidermal growth factor; EMT: Epithelial-to-mesenchymal transition; ER: Estrogen receptor; ET: PMC42-ET cells; FACS: Fluorescence-activated cell sorting; FCS: Fetal calf serum; ICC: Immunocytochemistry; IF: Immunofluorescence; IHC: Immunohistochemistry; LA: PMC42-LA cells; LN MET: Lymph node metastatic; MET: Mesenchymal-to-epithelial transition; MT-PCR: Multiplex tandem polymerase chain reaction; PBS: Phosphate-buffered saline; QRT-PCR: Quantitative reverse-transcription polymerase chain reaction; RPMI: Roswell Park Memorial Institute medium; shRNA: Short-hairpin ribonucleic acid; SRB: Sulforhodamine B; WT: Wild type.

## Competing interests

The authors declare that they have no competing interests.

## Authors’ contributions

HJH conceived the study design and performed all experiments not attributed to others. LP performed chromatin immunoprecipitation studies, Western blot analysis (Figure 4B), and provided support for the CAT reporter analyses; RS and BS carried out cell-cycle analyses and interpretation; YD and TJG created the MYB-modified MCF-7 cell line; CP performed the EpCAM bead-binding assay and EpCAM FACS analyses; CP, ETLS, BvD, and DFN performed cell-migration assays and/or analyses. DG contributed to the experiments with MDA-MB-468 cells; PH assisted in histopathologic interpretations and provided archival sections of breast cancers and matched lymph node metastases; HJH RGR, TJG, DFN, and EWT participated in the study design, coordination, and data interpretation. All authors participated in drafting the manuscript, and read and approved the final version.

## Authors’ information

HJH was supported by a National Breast Cancer Foundation Postdoctoral Training Fellowship; and EWT and HJH were supported also by the Cancer Council of Victoria; NPADG was supported by The Australian Government’s Endeavour Awards Scholarship Program; BvD and ETLS were supported by the National Breast Cancer Foundation (Australia) and Cancer Australia; EWT, DFN, TJG and CP are active members of the EMP*athy* Breast Cancer Network, a National Breast Cancer Foundation (Australia)-funded National Collaborative Research Program. This study benefited from support by the Victorian Government’s Operational Infrastructure Support Program to St. Vincent’s Institute and Murdoch Children’s Research Institute.

## Supplementary Material

Additional file 1 **(A)** The expression (MT-PCR) of other EMT-related genes in SCRsh-ET versus ZEB1sh-ET, not shown in Figure 4** (A, part iii). (B)** Additional SRB experiments supporting the SRB data shown in Figure 4C, part **ii**. **(C)** Clonogenic assay of SCRsh-ET versus ZEB1sh-ET showing that ZEB1 knockdown led to larger overall colony size, consistent with a higher rate of proliferation. Colony number and size were averaged from thresholded images of six individual 10-cm-diameter plates (*n* = 6) by using the Image J image-analysis program (*P < 0.05; error bars represent SEM. Statistics were determined by using Student paired *t* test). Click here for file

Additional file 2 **MYB-targeted knockdown in ZEB1sh-ET cells abrogates the effect of ZEB1KD on colony size. (A) (i)** Validation of MYB knockdown in MYB shRNA expressing SCRsh-ET and ZEB1sh-ET cells by QRT-PCR (fold expression relative to SCRsh-ET/SCRsh-ET shown, *n* = 1, error bars represent SD) and **(ii)** Western blotting analysis. **(B)** clonogenicity assay revealing ZEB1sh/MYBsh-ET cells form smaller colonies, indicating a reduction in proliferative rate (*n* = 3 independent biologic replicates, error bars represent SEM). Click here for file

Additional file 3 **(A)** DAB immunocytochemistry staining of ZEB1sh-ET shows reduced nuclear ZEB1, magnification 600×, scale bar, 100 μm; **(B)** The EpCAM receptor was reexpressed in ZEB1sh-ET, as shown by **(i)** greater retention to EpCAM coated beads (inferred by reduced number of unbound cells in the flow-through population); and **(ii)** FACS for EpCAM. CDH1 protein reexpression shown in Figure 4A was further confirmed by FACS to be membrane bound. The results from three independent experiments (biologic replicates) are shown. **(C) (i)** Confocal microscopy of PMC42-ET spheroids grown on Matrigel revealed CDH1 was expressed at the cell membrane in ZEB1sh-ET, magnification 400×, scale bar, 30 μ*M*; **(ii)** ZEB1sh-ET spheroids were generally larger than SCRsh-ET, consistent with previous work in MDA-MB-231, in which ZEB1 was knocked down [10]. PMC42-ET WT cell organoids were not enlarged and were comparable in size to SCR shRNA-ET control (not shown). 100× magnification; scale bar, 200 μ*M*. **(iii)** ZEB1sh-ET cells grew as tighter clusters in 2D culture (magnification 200×; scale bar, 50 μ*M*) and exhibited slower epithelial-like movement in this dimension (see Additional file 4). **(D)** MYB was expressed higher in ZEB1sh-ET cells, regardless of the dimension of culture. Click here for file

Additional file 4 **PMC42 cells transfected with siRNA: scrambled (A) or Zeb1 (B).** Stills from movies at an interval of 3 hours are shown, and paths of movement of seven cells in each field are projected in yellow on the first time frame of each. Traces for PMC42ET-shSCR control (147.3 ± 52.1 μm/4 hours) and PMC42ET-shZeb1 cells (60.9 ±17.2 μm/4 hours; *P* < 0.0001). Note that the more-epithelial appearance of the Zeb1 siRNA-transfected cells correlates with very short paths. Tracking point was the center of the cell nucleus. Click here for file

Additional file 5 **(A)** Immunofluorescence of MDA-MB-468 cells seeded in Terasaki plates exposed to hypoxic conditions (1% O_2_) for various times, as indicated. CDH1 sublocalization to the cytoplasm from the membrane was seen, along with an increase in the expression of VIM protein in a time-dependent manner on exposure to hypoxia; magnification 600×; scale bar, 50 μm. **(B)** Phase-contrast images of untreated or 10 ng/ml EGF-treated PMC42-LA, depicting the acquisition of mesenchymal features: scattering and an elongation of cell shape, magnification 100×; scale bar, 200 μ*M*. **(C)** Comparison of expression levels of MYB, ZEB1, and CDH1 in Luminal + BasalA versus BasalB subgroups of human breast cancer cells from the Neve dataset [13]. Significance (*) set at *P* < 0.05, Mann–Whitney statistical test. **(D)** An additional matched human breast tumor primary (Primary T) and sentinel lymph node metastases (LN MET) set to that shown in Figure 5; magnification, 400×; scale bar, 150 μm. Click here for file

Additional file 6 **Additional cell models of EMT in which MYB was repressed (A, B). (A)** Expression (QRT-PCR) analyses of MCF-7 cells exposed to hypoxia for up to 72 hours. **(B)** T47D cells treated for up to 120 hours with 10 ng/ml EGF. Click here for file

Additional file 7 **Representative images of the (A) vector control and (B) MYB-transfected MDA-MB-231 cells under phase-contract microscopy, showing tracking of path length over a 4–hour period.** For each, two groups of seven closely spaced cells were selected for tracking. Tracking point was the center of the cell nucleus. Yellow denotes representative 4-hour traces for MDA-MB-231-vector control (147.7 ± 30.6 μm/4 hours; 4-hour displacement = 42% of path length, that is, relatively straight) and MYB-transfected MDA-MB-231 cells (91.2 ±19.01 μm/4 hours; 4-hour displacement = 25% of path length; that is, quite tortuous). Click here for file

Additional file 8 **(A)** Knockdown of MYB in PMC42-LA cells (which were characterized in Figure 1) led to a further reduction of CDH1 expression after induction of EMT by EGF. Data shown are fold change, relative to untreated SCRsh-LA; *n* = 1, error bars represent standard deviation. **(B)** Stable overexpression of MYB in MCF-7 cells **(i)** validated by immunocytochemistry (DAB), magnification 100×, scale bar, 200 μm; and **(ii)** fold changes in mesenchymal gene expression compared with WT MCF-7. Data shown are an average of three independent experiments; error bars represent SEM, significance (*P* < 0.05) indicated by *, as determined by Student paired *t* test. **(C)** Stable MYB overexpression in MDA-MB-231 cells does not result in Snail2 expression upregulation: MT-PCR data expressed as fold change (Vector control MDA-MB-231 = 1); *n* = 1; error bars represent standard deviation. Click here for file

Additional file 9 **(A)** E and Z-boxes number1 within the MYB promoter captured in the ChIP analysis are highly conserved between *Homo sapiens*, *Mus muscularis,* and *Canis familiaris*, as are E and Z-boxes number 2 downstream. **(B)** Additional graphs/replications of ChIP analyses summarized in Figure 2: QRT-PCR of two independent replications of ChIP assay examining the region of the MYB promoter defined in Figure 1. **(C)** QRT-PCR positive control for ChIP assay: amplification of the region of the CDH1 promoter containing E-boxes at which ZEB1 has been demonstrated to bind. **(D)** QRT-PCR negative control: amplification of a nonrelated sequence within MYB gene in intron 10. Click here for file

Additional file 10 **An *inverse* correlation was observed between Snail2 and MYB expression in Luminal versus BasalB subgroups of human breast cancer cell lines from the Neve dataset **[13]**.** Significance (*) set at *P* < 0.05, Mann–Whitney statistical test. Click here for file
