# Supplementary figures and images for: Direct repression of MYB by ZEB1 suppresses proliferation and epithelial gene expression during epithelial-to-mesenchymal transition of breast cancer cells
Source: Breast Cancer Res. 2013 Nov 27;15(6):R113. doi: 10.1186/bcr3580 (PMC3979034; doi:10.1186/bcr3580)

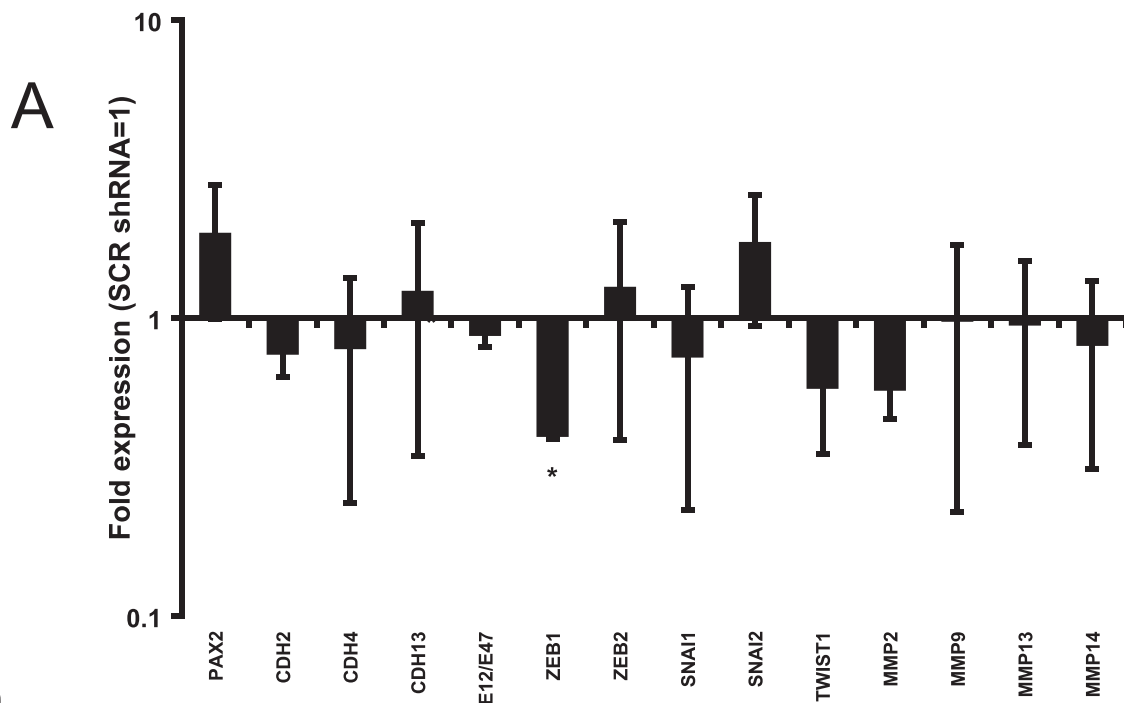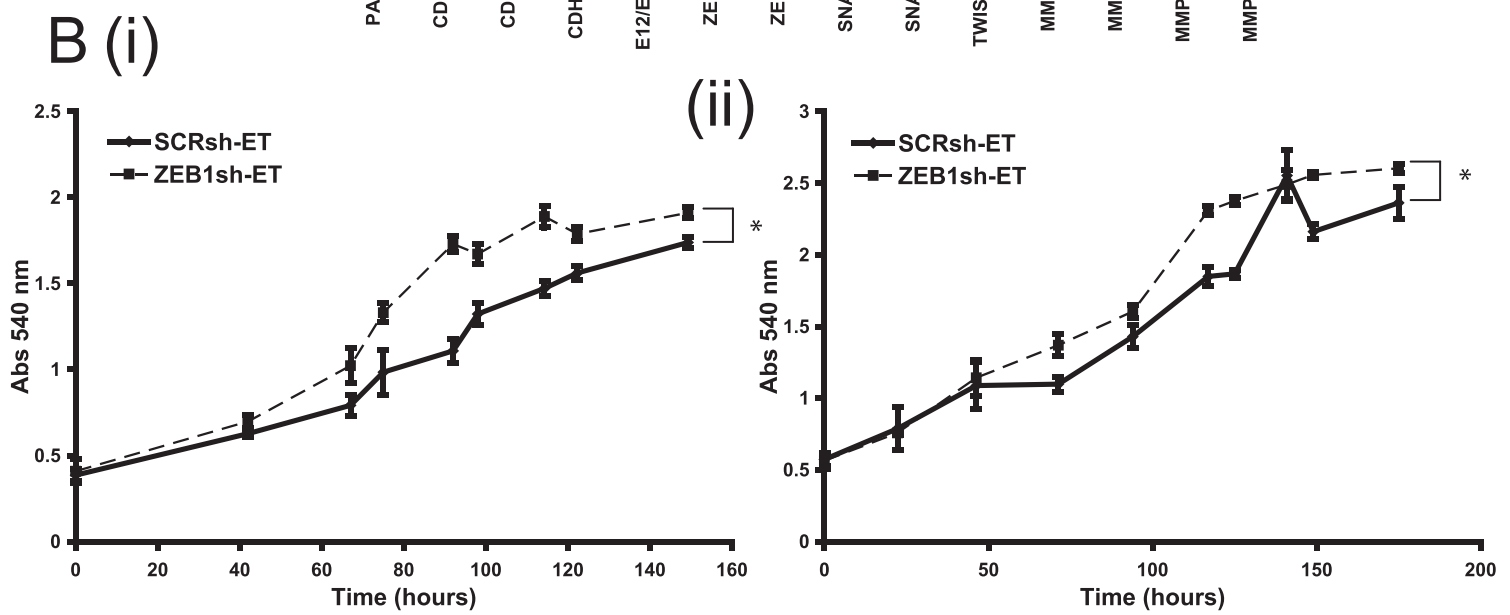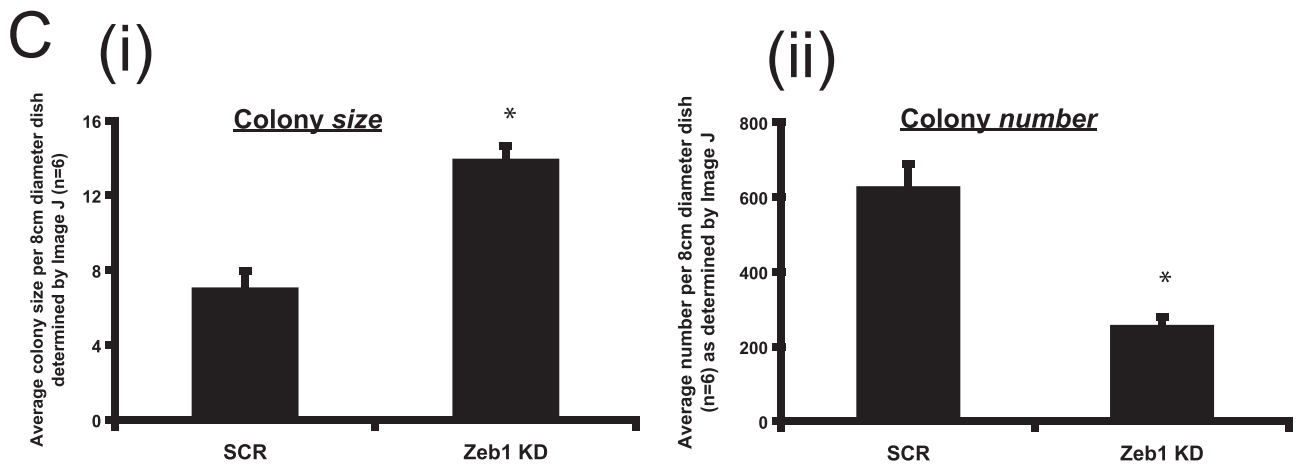

**(iii)**

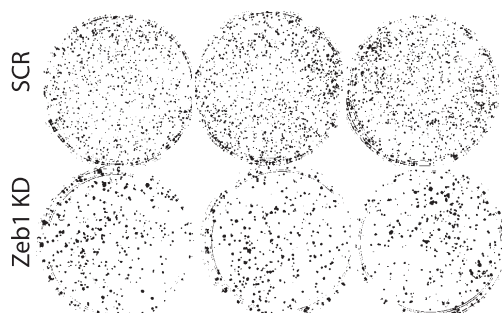

Additional  
file 1

Supplement: Additional file 1 — (A) The expression (MT-PCR) of other EMT-related genes in SCRsh-ET versus ZEB1sh-ET, not shown in Figure 4 (A, part iii). (B) Additional SRB experiments supporting the SRB data shown in Figure 4C, part ii. (C) Clonogenic assay of SCRsh-ET versus ZEB1sh-ET showing that ZEB1 knockdown led to larger overall colony size, consistent with a higher rate of proliferation. Colony number and size were averaged from thresholded images of six individual 10-cm-diameter plates (n = 6) by using the Image J image-analysis program (*P < 0.05; error bars represent SEM. Statistics were determined by using Student paired t test). [file bcr3580-S1.pdf]

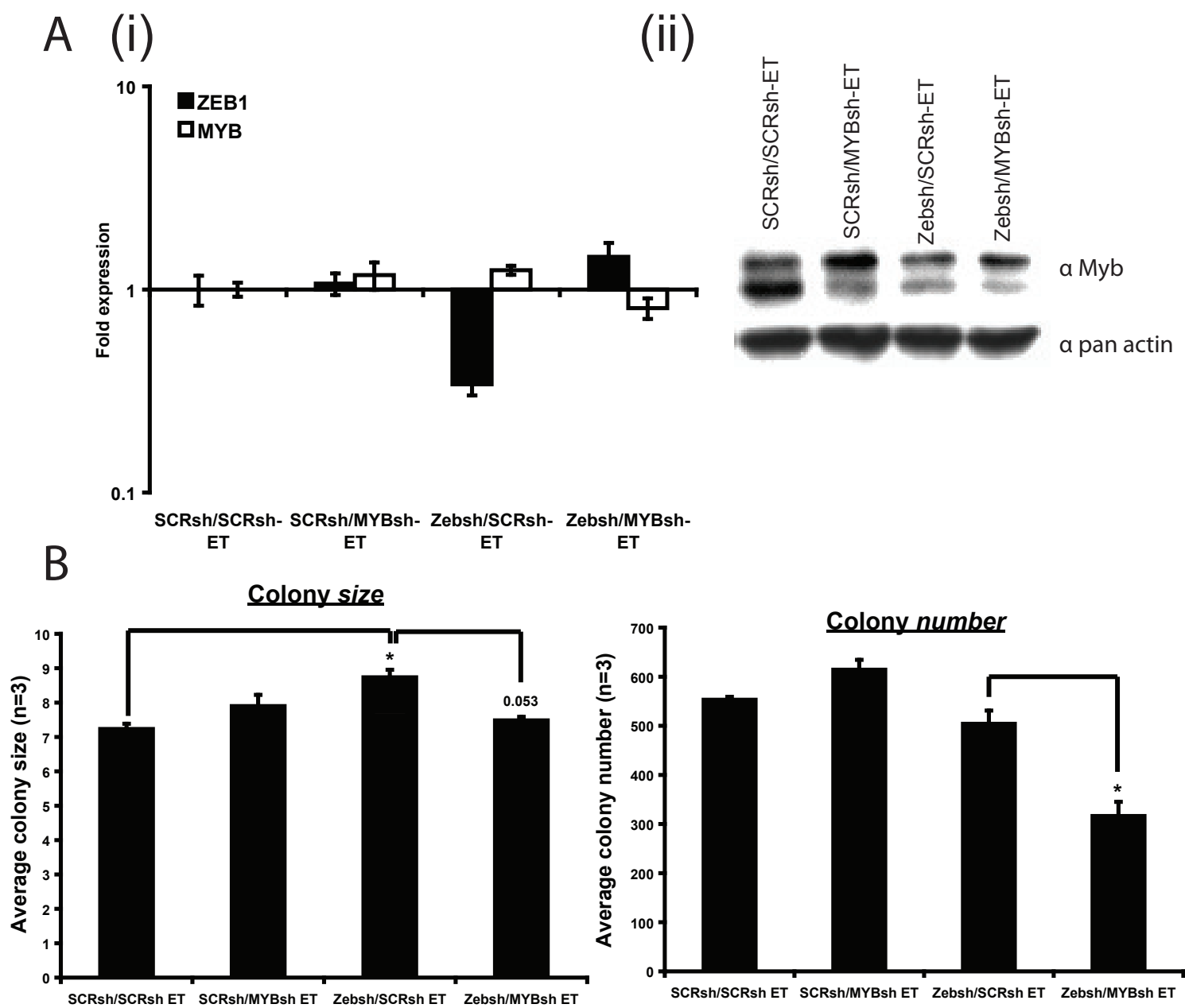

Additional file 2

Supplement: Additional file 2 — MYB-targeted knockdown in ZEB1sh-ET cells abrogates the effect of ZEB1KD on colony size. (A) (i) Validation of MYB knockdown in MYB shRNA expressing SCRsh-ET and ZEB1sh-ET cells by QRT-PCR (fold expression relative to SCRsh-ET/SCRsh-ET shown, n = 1, error bars represent SD) and (ii) Western blotting analysis. (B) clonogenicity assay revealing ZEB1sh/MYBsh-ET cells form smaller colonies, indicating a reduction in proliferative rate (n = 3 independent biologic replicates, error bars represent SEM). [file bcr3580-S2.pdf]

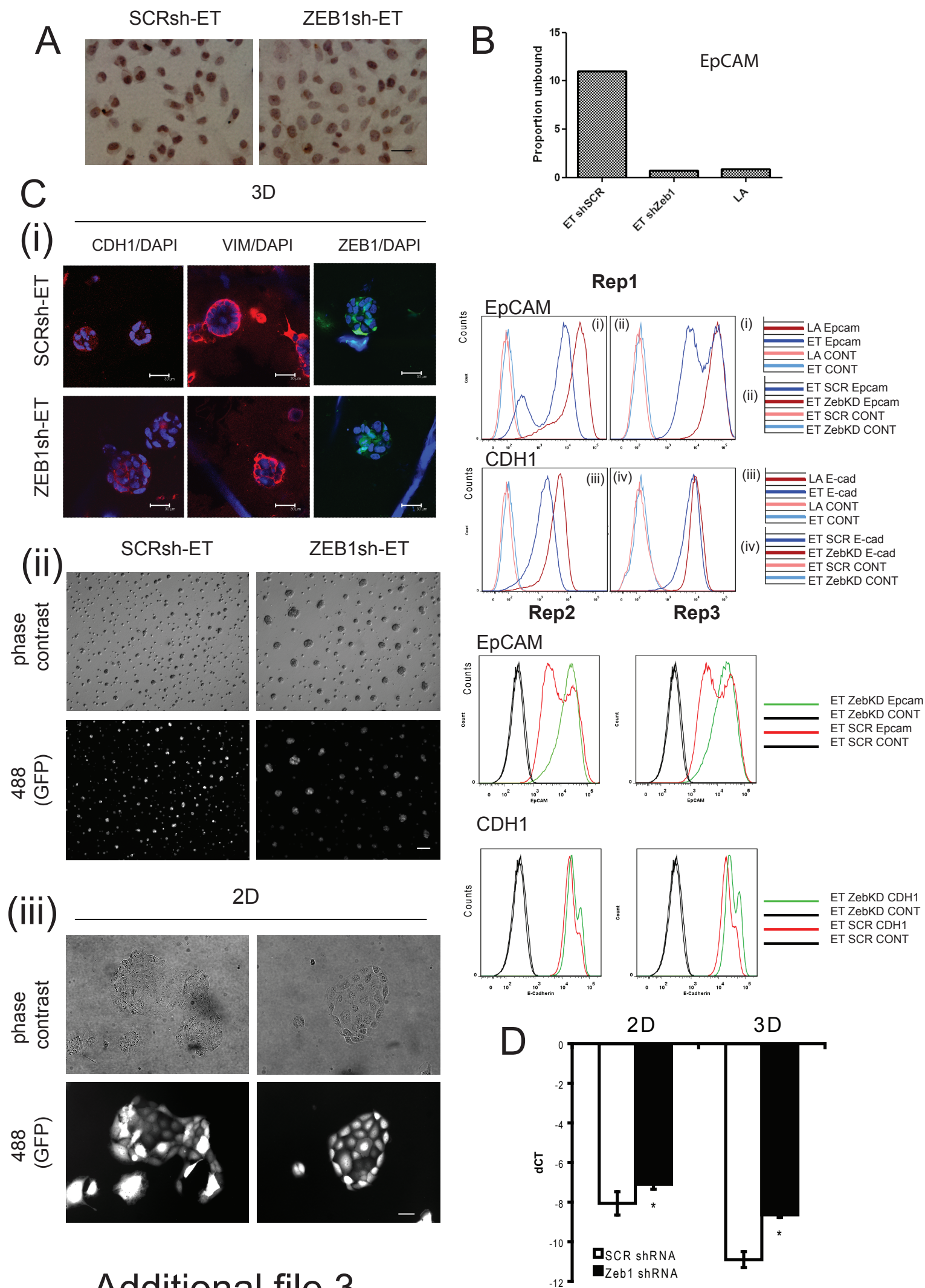

Supplement: Additional file 3 — (A) DAB immunocytochemistry staining of ZEB1sh-ET shows reduced nuclear ZEB1, magnification 600×, scale bar, 100 μm; (B) The EpCAM receptor was reexpressed in ZEB1sh-ET, as shown by (i) greater retention to EpCAM coated beads (inferred by reduced number of unbound cells in the flow-through population); and (ii) FACS for EpCAM. CDH1 protein reexpression shown in Figure 4A was further confirmed by FACS to be membrane bound. The results from three independent experiments (biologic replicates) are shown. (C) (i) Confocal microscopy of PMC42-ET spheroids grown on Matrigel revealed CDH1 was expressed at the cell membrane in ZEB1sh-ET, magnification 400×, scale bar, 30 μM; (ii) ZEB1sh-ET spheroids were generally larger than SCRsh-ET, consistent with previous work in MDA-MB-231, in which ZEB1 was knocked down [10]. PMC42-ET WT cell organoids were not enlarged and were comparable in size to SCR shRNA-ET control (not shown). 100× magnification; scale bar, 200 μM. (iii) ZEB1sh-ET cells grew as tighter clusters in 2D culture (magnification 200×; scale bar, 50 μM) and exhibited slower epithelial-like movement in this dimension (see Additional file 4). (D) MYB was expressed higher in ZEB1sh-ET cells, regardless of the dimension of culture. [file bcr3580-S3.pdf]

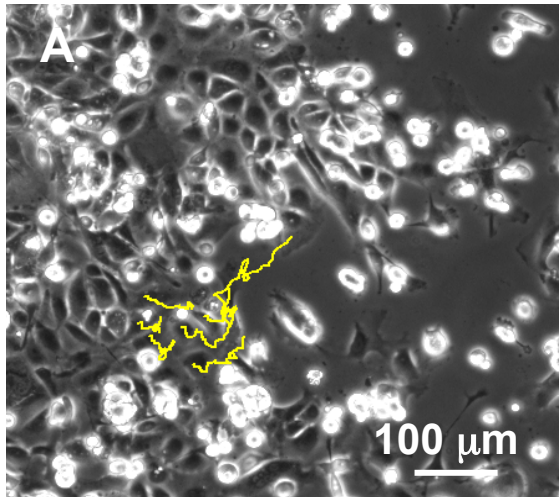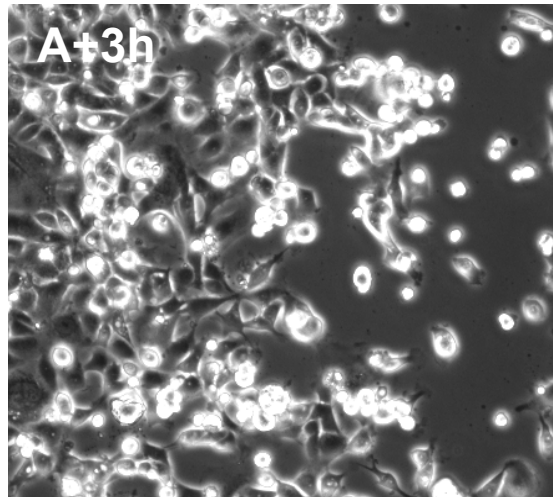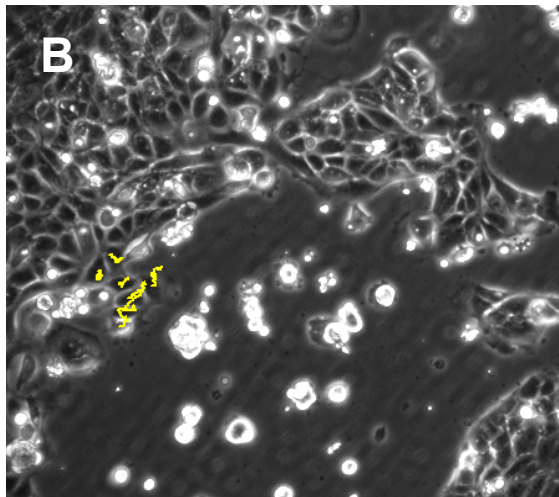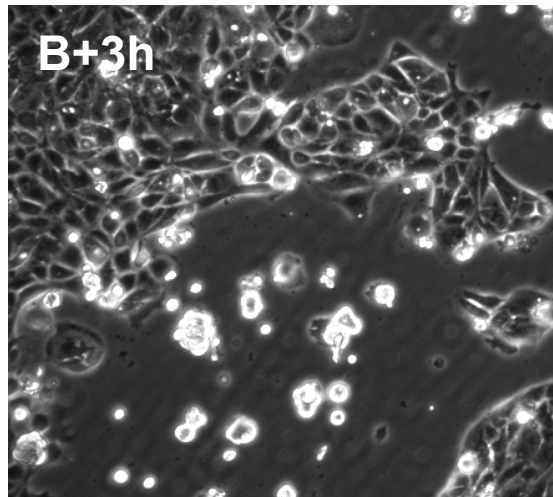

Supplement: Additional file 4 — PMC42 cells transfected with siRNA: scrambled (A) or Zeb1 (B). Stills from movies at an interval of 3 hours are shown, and paths of movement of seven cells in each field are projected in yellow on the first time frame of each. Traces for PMC42ET-shSCR control (147.3 ± 52.1 μm/4 hours) and PMC42ET-shZeb1 cells (60.9 ±17.2 μm/4 hours; P < 0.0001). Note that the more-epithelial appearance of the Zeb1 siRNA-transfected cells correlates with very short paths. Tracking point was the center of the cell nucleus. [file bcr3580-S4.pdf]

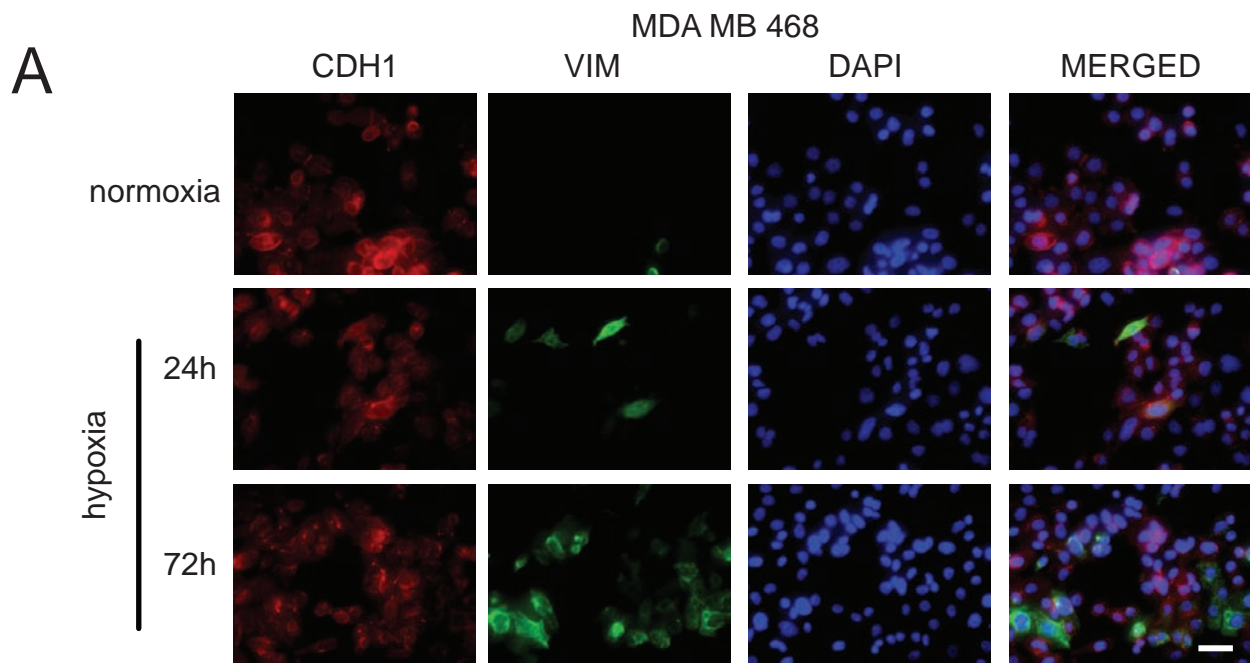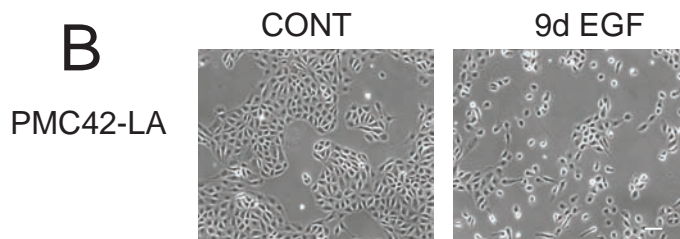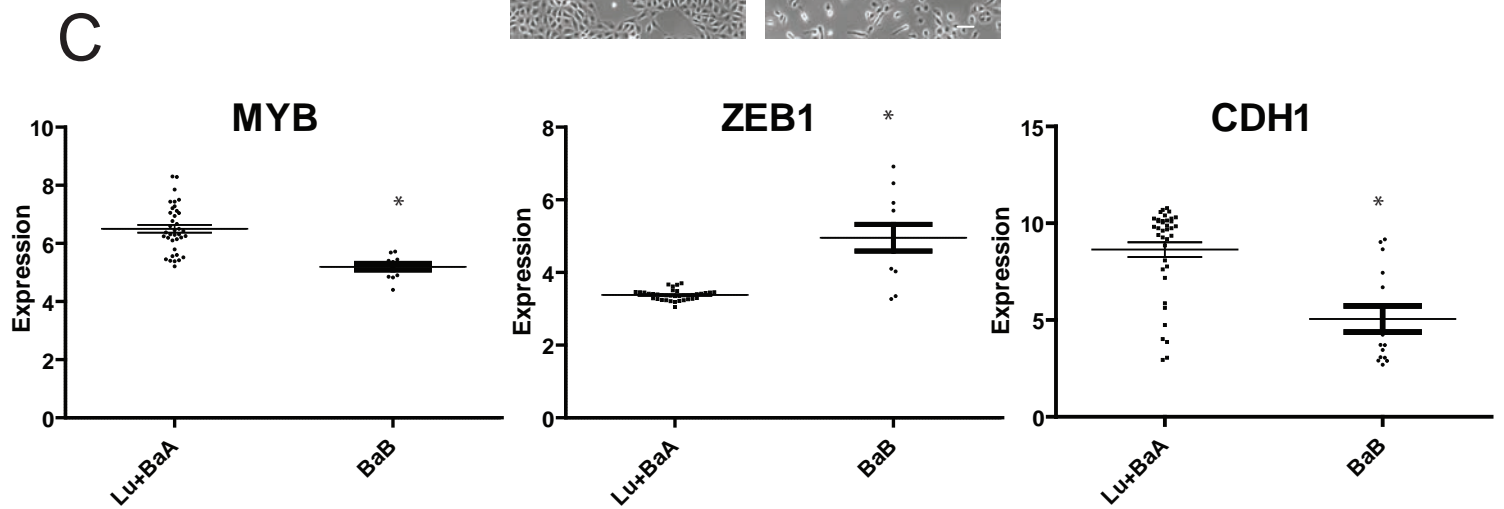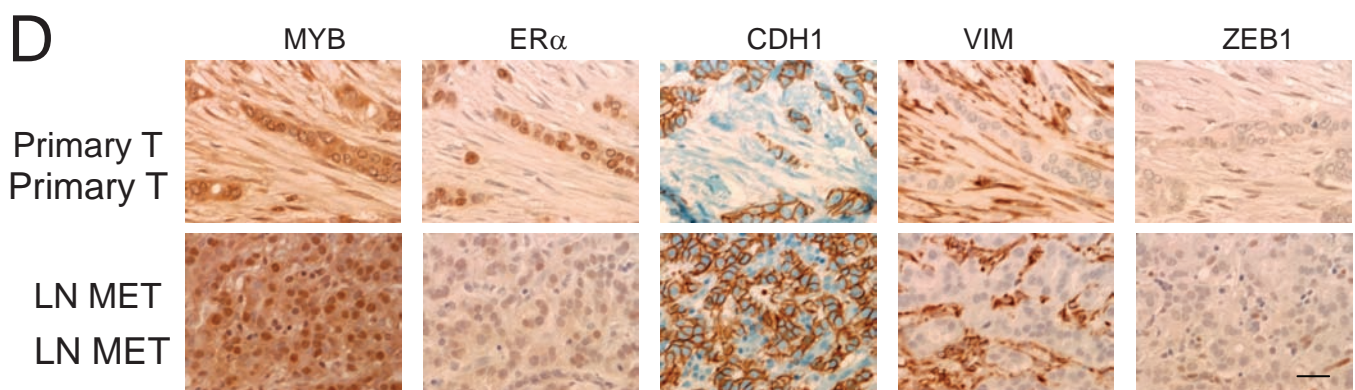

Supplement: Additional file 5 — (A) Immunofluorescence of MDA-MB-468 cells seeded in Terasaki plates exposed to hypoxic conditions (1% O2) for various times, as indicated. CDH1 sublocalization to the cytoplasm from the membrane was seen, along with an increase in the expression of VIM protein in a time-dependent manner on exposure to hypoxia; magnification 600×; scale bar, 50 μm. (B) Phase-contrast images of untreated or 10 ng/ml EGF-treated PMC42-LA, depicting the acquisition of mesenchymal features: scattering and an elongation of cell shape, magnification 100×; scale bar, 200 μM. (C) Comparison of expression levels of MYB, ZEB1, and CDH1 in Luminal + BasalA versus BasalB subgroups of human breast cancer cells from the Neve dataset [13]. Significance (*) set at P < 0.05, Mann–Whitney statistical test. (D) An additional matched human breast tumor primary (Primary T) and sentinel lymph node metastases (LN MET) set to that shown in Figure 5; magnification, 400×; scale bar, 150 μm. [file bcr3580-S5.pdf]

A

MCF-7

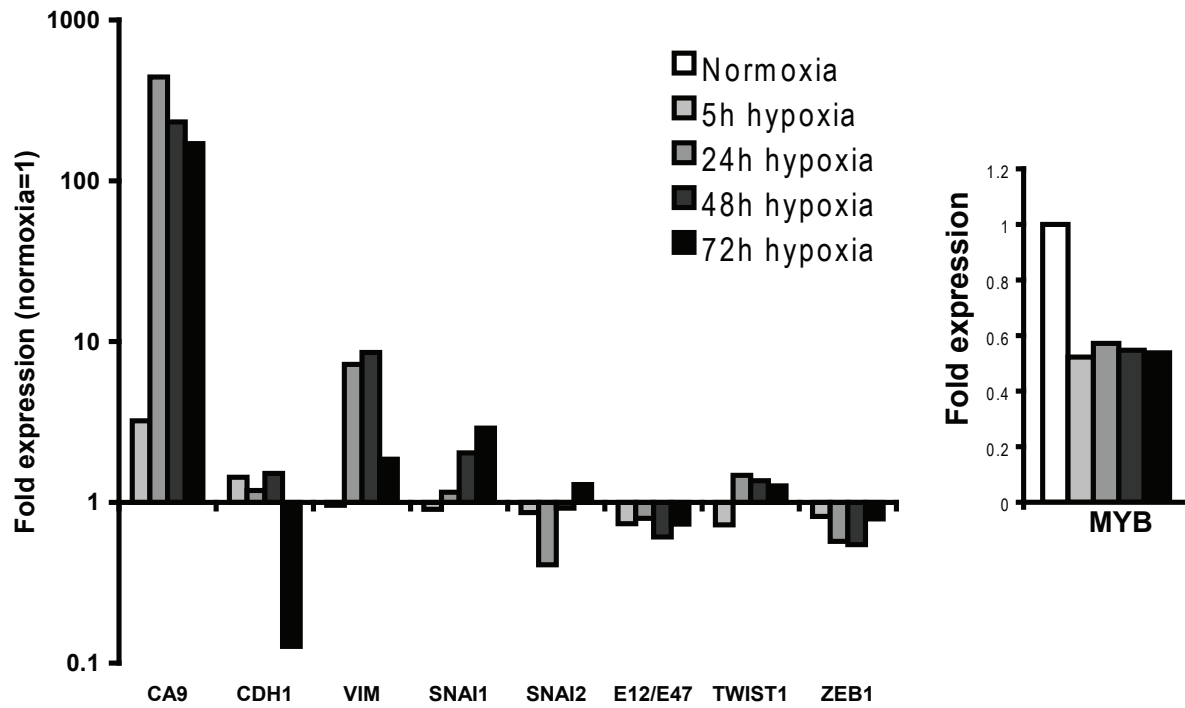

B

T47D

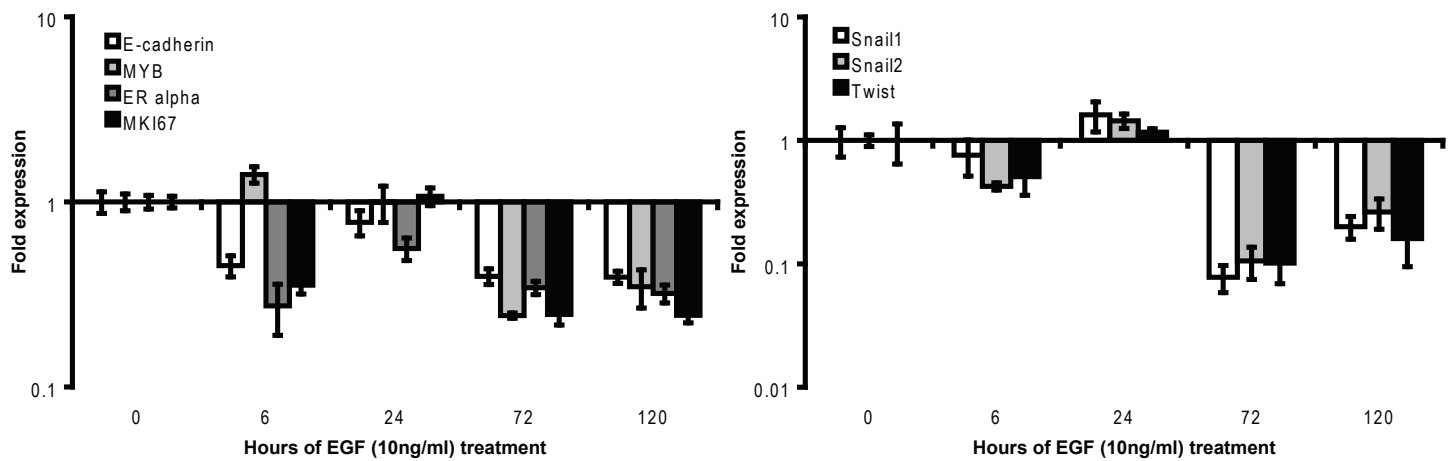

Additional file 6

Supplement: Additional file 6 — Additional cell models of EMT in which MYB was repressed (A, B). (A) Expression (QRT-PCR) analyses of MCF-7 cells exposed to hypoxia for up to 72 hours. (B) T47D cells treated for up to 120 hours with 10 ng/ml EGF. [file bcr3580-S6.pdf]

A

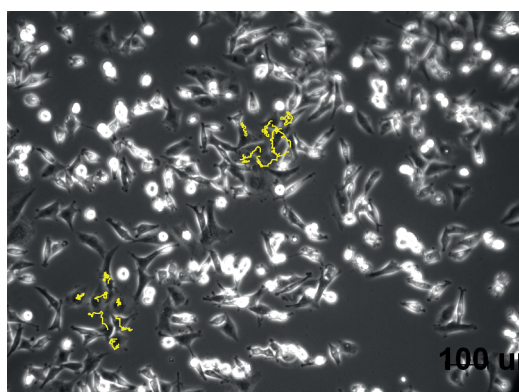

B

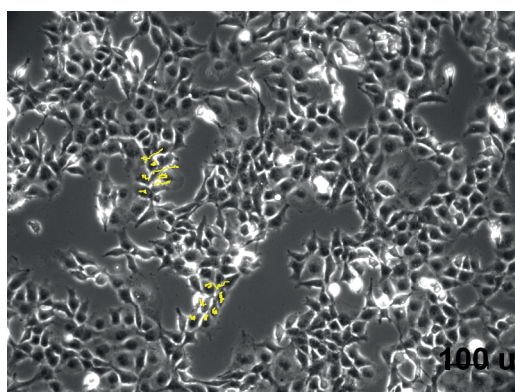

Additional file 7

Supplement: Additional file 7 — Representative images of the (A) vector control and (B) MYB-transfected MDA-MB-231 cells under phase-contract microscopy, showing tracking of path length over a 4–hour period. For each, two groups of seven closely spaced cells were selected for tracking. Tracking point was the center of the cell nucleus. Yellow denotes representative 4-hour traces for MDA-MB-231-vector control (147.7 ± 30.6 μm/4 hours; 4-hour displacement = 42% of path length, that is, relatively straight) and MYB-transfected MDA-MB-231 cells (91.2 ±19.01 μm/4 hours; 4-hour displacement = 25% of path length; that is, quite tortuous). [file bcr3580-S7.pdf]

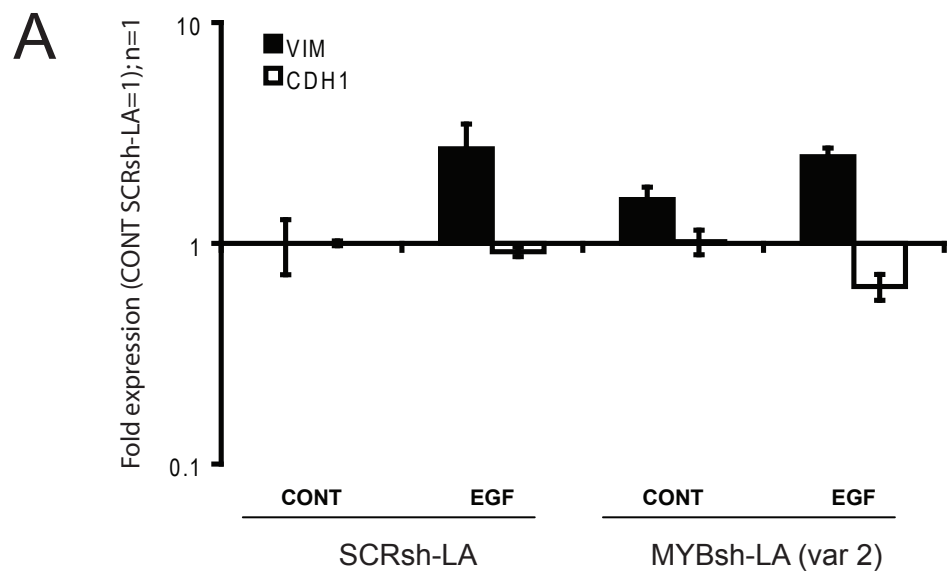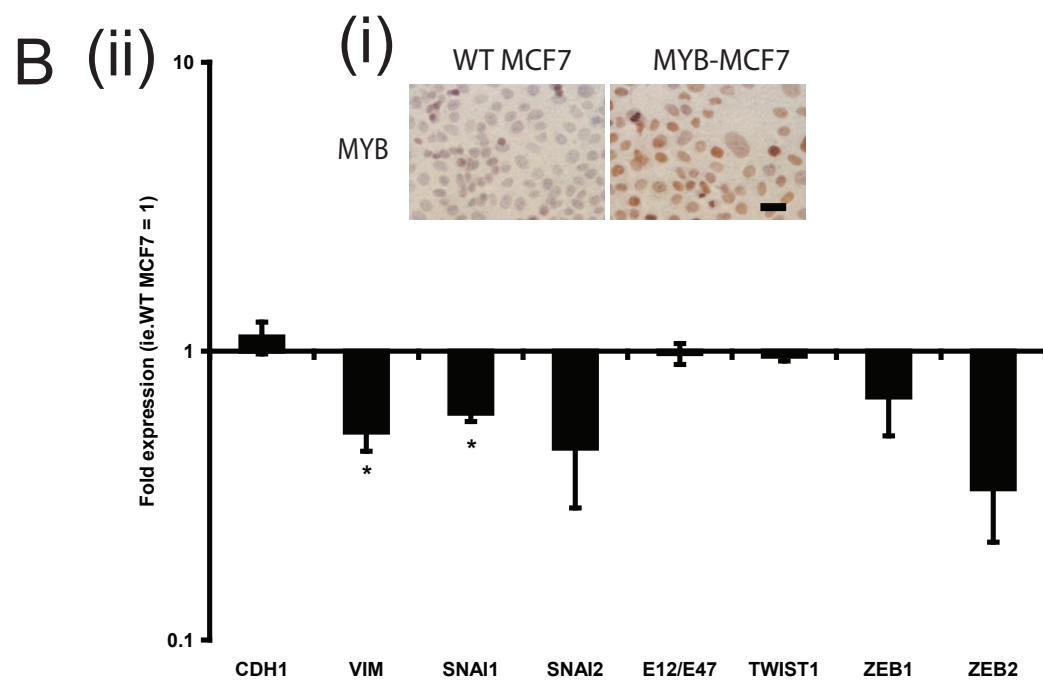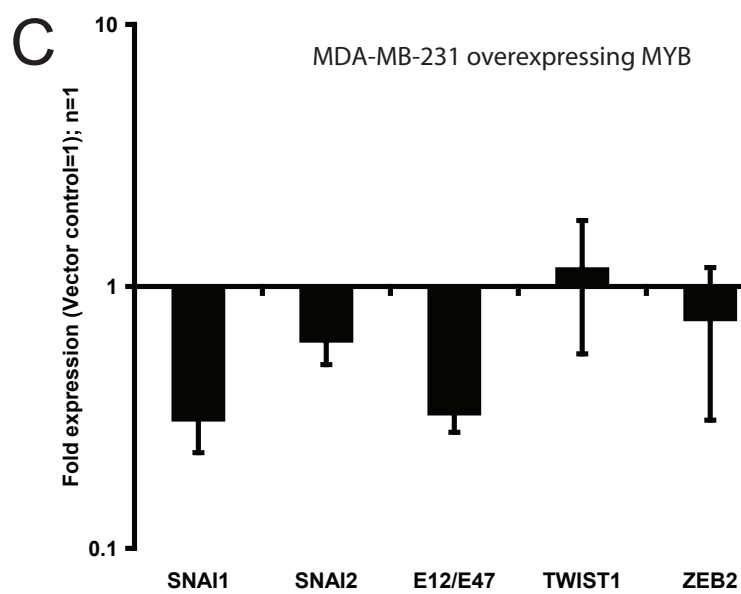

Supplement: Additional file 8 — (A) Knockdown of MYB in PMC42-LA cells (which were characterized in Figure 1) led to a further reduction of CDH1 expression after induction of EMT by EGF. Data shown are fold change, relative to untreated SCRsh-LA; n = 1, error bars represent standard deviation. (B) Stable overexpression of MYB in MCF-7 cells (i) validated by immunocytochemistry (DAB), magnification 100×, scale bar, 200 μm; and (ii) fold changes in mesenchymal gene expression compared with WT MCF-7. Data shown are an average of three independent experiments; error bars represent SEM, significance (P < 0.05) indicated by *, as determined by Student paired t test. (C) Stable MYB overexpression in MDA-MB-231 cells does not result in Snail2 expression upregulation: MT-PCR data expressed as fold change (Vector control MDA-MB-231 = 1); n = 1; error bars represent standard deviation. [file bcr3580-S8.pdf]

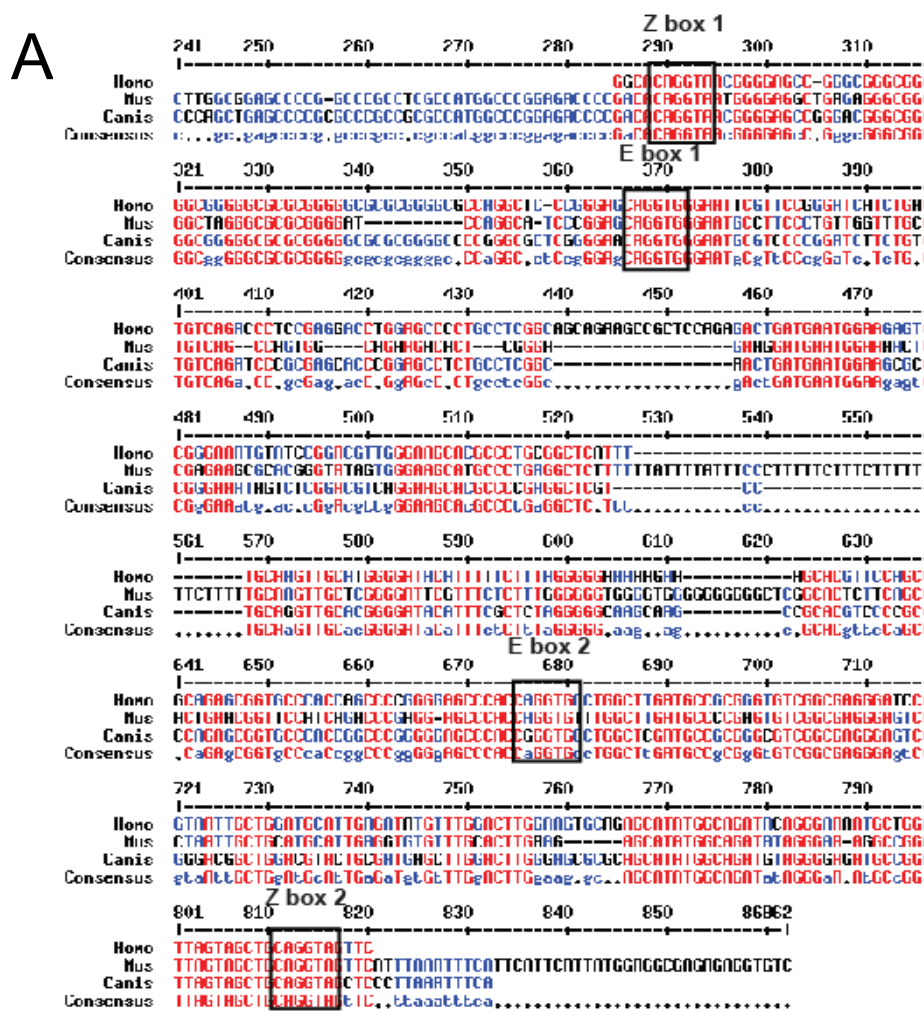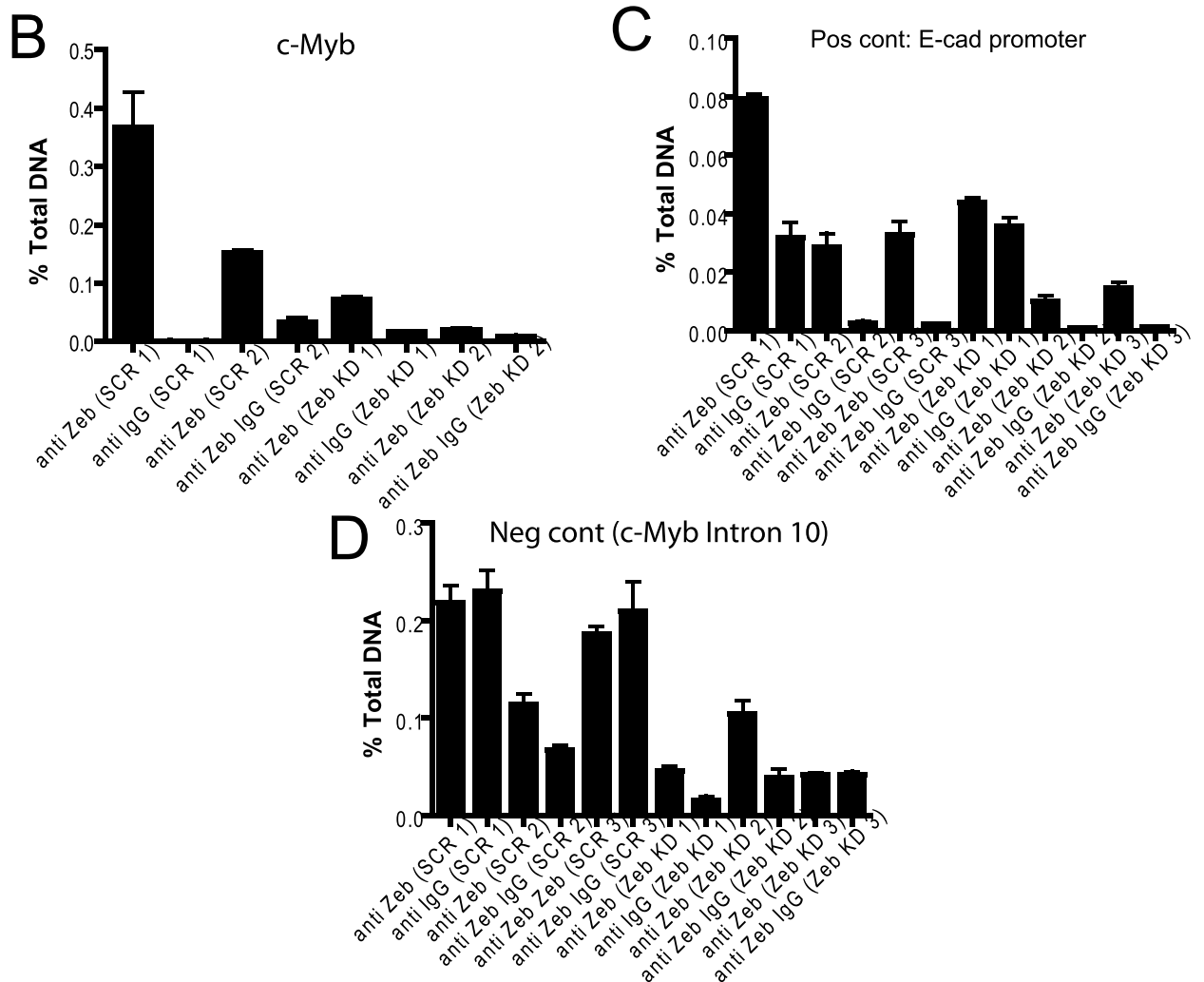

Supplement: Additional file 9 — (A) E and Z-boxes number1 within the MYB promoter captured in the ChIP analysis are highly conserved between Homo sapiens, Mus muscularis, and Canis familiaris, as are E and Z-boxes number 2 downstream. (B) Additional graphs/replications of ChIP analyses summarized in Figure 2: QRT-PCR of two independent replications of ChIP assay examining the region of the MYB promoter defined in Figure 1. (C) QRT-PCR positive control for ChIP assay: amplification of the region of the CDH1 promoter containing E-boxes at which ZEB1 has been demonstrated to bind. (D) QRT-PCR negative control: amplification of a nonrelated sequence within MYB gene in intron 10. [file bcr3580-S9.pdf]

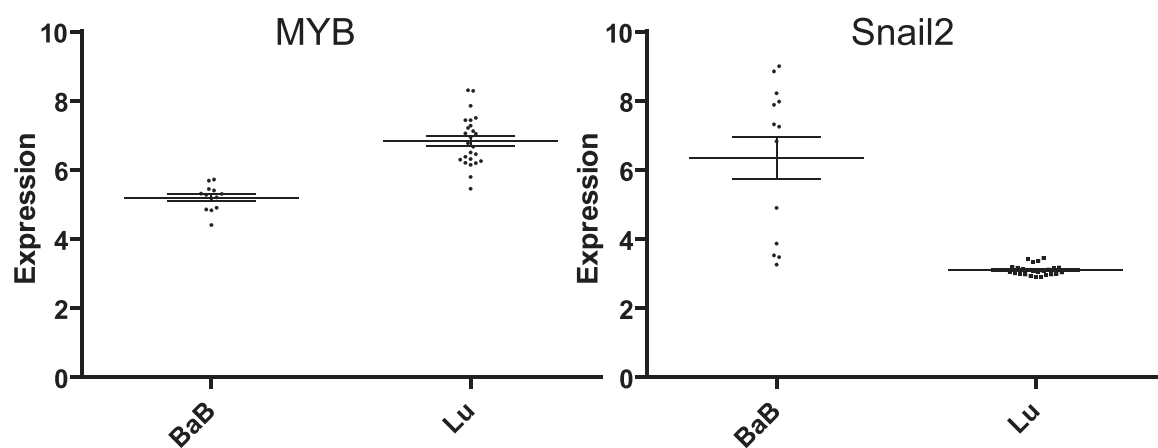

Additional file 10

Supplement: Additional file 10 — An inverse correlation was observed between Snail2 and MYB expression in Luminal versus BasalB subgroups of human breast cancer cell lines from the Neve dataset [13]. Significance (*) set at P < 0.05, Mann–Whitney statistical test. [file bcr3580-S10.pdf]
